# Mutant *RB1* enhances therapeutic efficacy of PARPis in lung adenocarcinoma by triggering the cGAS/STING pathway

**DOI:** 10.1172/jci.insight.165268

**Published:** 2023-11-08

**Authors:** Qi Dong, Tong Yu, Bo Chen, Mingyue Liu, Xiang Sun, Huiying Cao, Kaidong Liu, Huanhuan Xu, Yuquan Wang, Shuping Zhuang, Zixin Jin, Haihai Liang, Yang Hui, Yunyan Gu

**Affiliations:** 1 Department of Systems Biology, College of Bioinformatics Science and Technology, and; 2 Department of Biochemistry and Molecular Biology, Harbin Medical University, Harbin, China.; 3 Department of Pharmacology (State-Province Key Laboratories of Biomedicine-Pharmaceutics of China, Key Laboratory of Cardiovascular Research, Ministry of Education), College of Pharmacy, Harbin Medical University, Harbin, China.; 4 Shanghai Frontiers Science Research Center for Druggability of Cardiovascular noncoding RNA, Institute for Frontier Medical Technology, Shanghai University of Engineering Science, Shanghai, China.

**Keywords:** Genetics, Therapeutics, Cancer immunotherapy, Drug therapy, Genetic instability

## Abstract

Poly (ADP-ribose) polymerase inhibitors (PARPis) are approved for cancer therapy according to their synthetic lethal interactions, and clinical trials have been applied in non–small cell lung cancer. However, the therapeutic efficacy of PARPis in lung adenocarcinoma (LUAD) is still unknown. We explored the effect of a mutated retinoblastoma gene (*RB1*) on PARPi sensitivity in LUAD. Bioinformatic screening was performed to identify PARPi-sensitive biomarkers. Here, we showed that viability of LUAD cell lines with mutated *RB1* was significantly decreased by PARPis (niraparib, rucaparib, and olaparib). *RB1* deficiency induced genomic instability, prompted cytosolic double-stranded DNA (dsDNA) formation, activated the cGAS/STING pathway, and upregulated downstream chemokines CCL5 and CXCL10, triggering immune cell infiltration. Xenograft experiments indicated that PARPi treatment reduced tumorigenesis in RB1-KO mice. Additionally, single-cell RNA sequencing analysis showed that malignant cells with downregulated expression of *RB1* had more communications with other cell types, exhibiting activation of specific signaling such as GAS, IFN response, and antigen-presenting and cytokine activities. Our findings suggest that *RB1* mutation mediates the sensitivity to PARPis through a synthetic lethal effect by triggering the cGAS/STING pathway and upregulation of immune infiltration in LUAD, which may be a potential therapeutic strategy.

## Graphical abstract

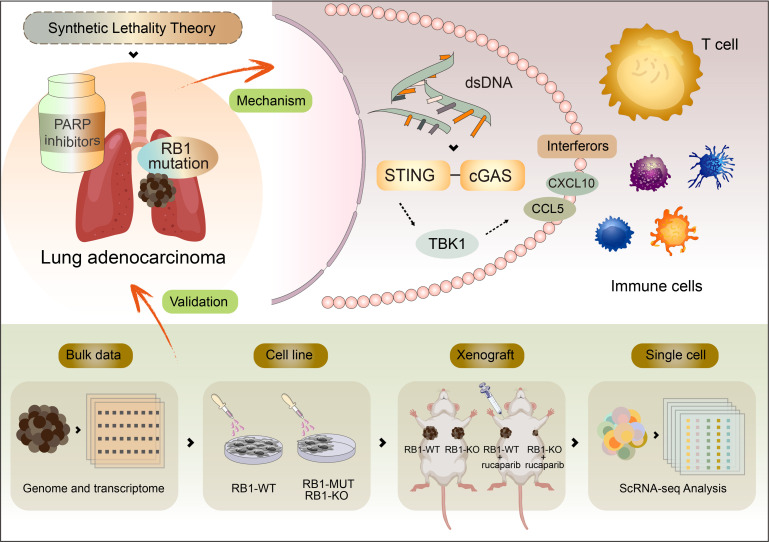

## Introduction

Lung adenocarcinoma (LUAD) is the major histological subtype of non–small cell lung carcinoma (NSCLC); the majority of diagnosed LUAD patients are in the middle and late stages (1). Despite promising new therapies that have been proposed, the 5-year survival rate of LUAD patients is 15% due to the typically late stage of diagnosis and innate or acquired resistance (2). Effective treatment strategies are needed for LUAD patients. Several clinical trials demonstrated that poly (ADP-ribose) polymerase inhibitors (PARPis) had the potential to improve the prognosis for NSCLC patients (3, 4). PARPis may therefore be useful treatments for LUAD patients.

PARPis are clinically approved drugs according to their synthetic lethality (SL) interaction, a phenomenon when 2 nonlethal genetic mutations emerge simultaneously that cause cancer cell death (5). PARP proteins, located in the nucleus, function as DNA damage receptors and signal transducers. PARP proteins regulate DNA single-strand-break repair by binding to damaged DNA and recruiting DNA-repair effector proteins (6). Patients with *BRCA1/2* mutations are sensitive to PARPis that induce cancer cell death by SL interaction (7). Recent studies showed that certain gene mutations interfere with the homologous recombination (HR) pathway or block DNA repair, inducing sensitivity to PARPis in cancer cells (8). A series of PARPis have been developed, such as rucaparib, veliparib, olaparib, and niraparib, which are applied for the treatment of breast, ovarian, pancreatic, and prostate cancers (9). However, there is a lack of effectively responsive biomarkers to PARPis in LUAD patients.

Genetic interactions refer to the combination of genetic variants of 2 genes contributing to a phenotype that is different from the expectation based on their individual variants, including SL and synthetic viability (10). Genetic interactions participate in the drug response of cancer cells. Hu et al. developed a quantitative chemical-genetic interaction mapping strategy in human mammary epithelial cells, charting the effect of knocking down 625 DNA repair– and cancer-relevant genes on the sensitivity or resistance to 29 drugs (11). Martins et al. generated a systematic chemical-genetic interaction map to predict 90 drug responses for triple-negative breast cancer (TNBC) cell lines with cancer gene aberrance (12). SL interactions contribute to the identification of new drug targets or drug response biomarkers.

Thus, we systematically explored candidate responsive biomarkers for PARPis based on genetic interactions by utilizing bioinformatics screening, and revealed that mutation of retinoblastoma tumor suppressor gene (*RB1*) is a candidate PARPi-sensitive biomarker for lung cancer (13). *RB1* is a negative regulator of the cell cycle. It has been shown that 8.2% of NSCLC patients carried mutations in *RB1*, and 4% of LUAD patients had mutations in *RB1* in The Cancer Genome Atlas (TCGA) (14). NSCLC patients with *RB1* mutation are associated with nonresponse to immunotherapy (15). Therefore, investigation of the role of *RB1* mutation in response to PARPis could be of great benefit for clinical treatment of NSCLC. Furthermore, the therapeutic response to PARPis is significantly influenced by the tumor microenvironment (TME) (16). Exploring and understanding the mechanism of the TME’s regulation of the response to PARPis is necessary for improving the effectiveness of treatment.

In the current study, we systematically explored the mechanism of *RB1* mutation involved in the response to PARPis (niraparib, rucaparib, and olaparib). Then, we investigated the effect of *RB1* mutation on the transcriptome and proteome. We found that *RB1* mutation increases genomic instability and upregulates the immune response by activating cGAS/STING signaling in LUAD. In vivo, rucaparib treatment significantly reduced tumor volume in *RB1*-knockout (RB1-KO) xenograft mice. Together, our results reveal that mutant *RB1* mediates sensitivity to PARPis in LUAD cells by SL interaction.

## Results

### RB1 mutation enhances sensitivity to PARPis in LUAD.

**Figure 1:**
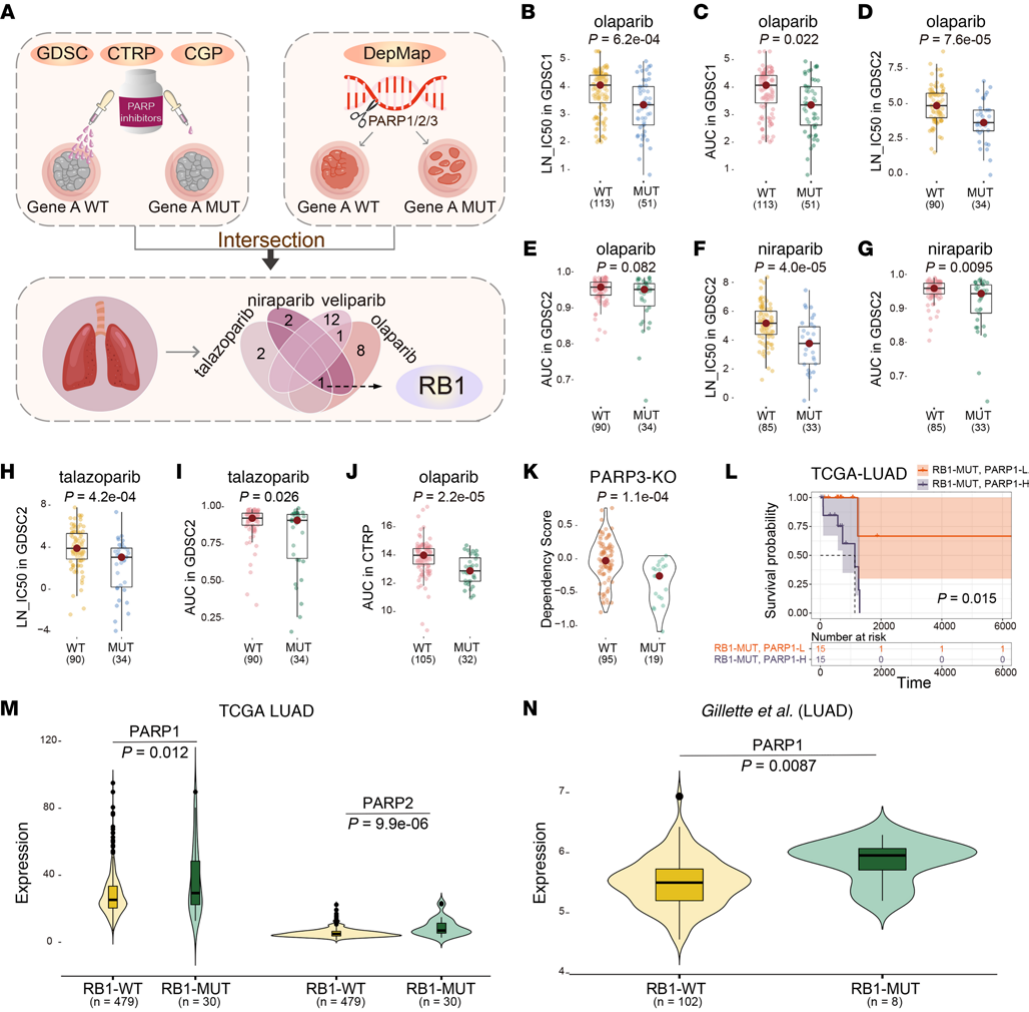
*RB1* mutation mediates sensitivity to PARPis. (**A**) Identification of *RB1* mutation as a candidate PARPi-sensitive biomarker. (**B**–**J**) Lung cancer cell lines with *RB1* mutations are sensitive to PARPis in the GDSC and CTRP data sets. LN_IC50, ln(IC_50_). (**K**) *RB1*-mutant lung cancer cell lines have lower viability when *PARP3* was knocked down in the DepMap (Achilles) data set. (**L**) Kaplan-Meier overall survival curves between patients with high and low *PARP1* expression with *RB1* mutations in the TCGA LUAD. (**M**) Distribution of *PARP1* and *PARP2* expression in cell lines with mutated *RB1* and WT *RB1* in TCGA data set. (**N**) Distribution of *PARP1* expression in cell lines with mutated *RB1* and WT *RB1* in the Gillette et al. data set (19). *P* values were calculated by 1-sided Wilcoxon’s rank-sum test (**B**–**K**, **M**, and **N**) and log-rank test (**L**).

Based on the systematic screening of genomic variations that have SL interactions with PARPis, we found that *RB1* mutation may mediate sensitivity to PARPis in lung cancer cells (Figure 1A) (13). *RB1*-mutant lung cancer cells tended to be sensitive to olaparib, niraparib, and talazoparib in the Genomics of Drug Sensitivity in Cancer (GDSC) and the Cancer Therapeutics Response Portal (CTRP) data sets (Figure 1, B–J). Moreover, *RB1* mutation had SL interaction with *PARP3* knockdown in the RNA interference screening data set (Cancer Dependency Map [DepMap] Portal, Achilles) (Figure 1K). In *RB1*-mutant LUAD patients, high expression of *PARP1* showed poorer overall survival than patients with low expression of *PARP1* (Figure 1L).

Then, we investigated the relationship between *RB1* mutation and *PARP1/2/3* expression. The expression of *PARP1* and *PARP2* in *RB1*-mutant LUAD samples was significantly higher than *RB1* wild-type (WT) samples (Figure 1, M and N). Furthermore, the expression of *PARP1*, *PARP2*, and *PARP3* was significantly negatively correlated with *RB1* expression (Supplemental Figure 1; supplemental material available online with this article; https://doi.org/10.1172/jci.insight.165268DS1).

**Figure 2:**
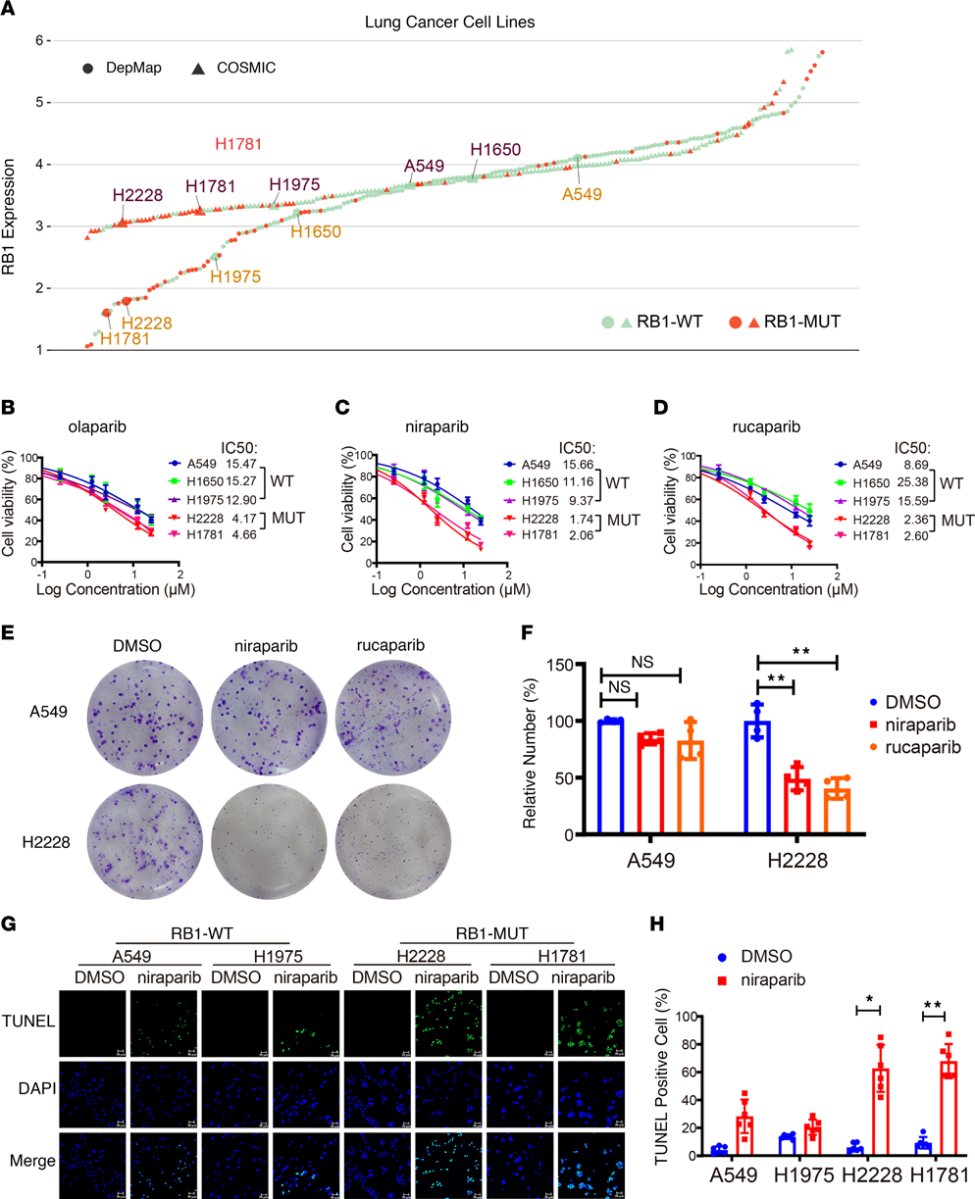
LUAD cell lines with *RB1* mutations reduce viability promoted by PARPis. (**A**) Expression and mutations of *RB1* in 5 LUAD cells in the DepMap and COSMIC databases. (**B**–**D**) The viability of LUAD cell lines with mutated *RB1* or WT *RB1* treated with PARPis olaparib, niraparib, and rucaparib were analyzed by CCK8 assay (*n* = 4). (**E**) Representative images of colony formation assay of A549 and H2228 cell lines treated with 1 μM niraparib, rucaparib, and DMSO for 72 hours followed by 14-day recovery. (**F**) Statistical analysis of colony formation assay (*n* = 4). **P < 0.01 vs. DMSO by unpaired, 2-tailed Student’s *t* test and Dunnett’s test. NS, no significance. (**G**) TUNEL assay of apoptotic cells among A549, H1975, H2228, and H1781 treated with 1 μM DMSO or niraparib. Scale bars: 50 μm. (**H**) Statistical analysis of TUNEL assay (*n* = 6). *P < 0.05, **P < 0.01 vs. DMSO by unpaired, 2-tailed Student’s *t* test.

H2228 and H1781 are *RB1*-mutant cell lines, whereas A549, H1650, and H1975 are *RB1*-WT cell lines (Figure 2A). CCK8 analysis showed that *RB1*-mutant cell lines were sensitive to PARPis (Figure 2, B–D). Consistent with these results, the IC_50_ values of the LUAD cells were significantly lower in cell lines with *RB1* mutation compared with WT cell lines (Figure 2, B–D). Furthermore, H2228 formed fewer colonies than A549 when exposed to the same concentration of niraparib and rucaparib for the same time (Figure 2, E and F). TUNEL assay showed that *RB1-*mutant cell lines H2228 and H1781 had more apoptotic cells compared with WT cell lines A549 and H1975 treated with niraparib. Cells treated with DMSO served as the negative control (Figure 2, G and H). The results indicate that *RB1* mutation increases the sensitivity to PARPis in LUAD cells.

### Mutated RB1 shows low expression at the level of transcriptome and proteome and mediates sensitivity to PARPis.

**Figure 3:**
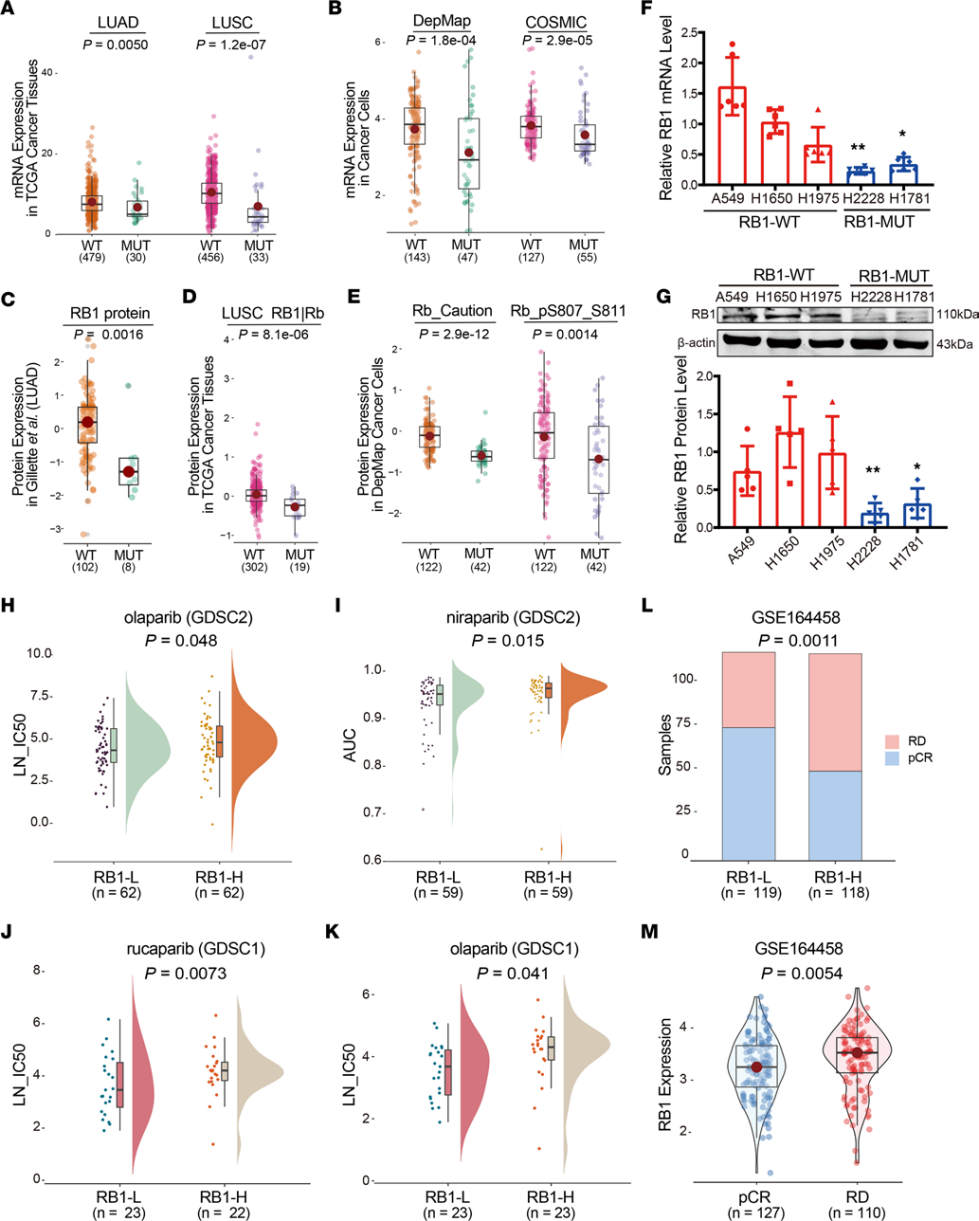
Loss-of-function mutations of *RB1*. (**A** and **B**) Distribution of *RB1* mRNA expression in cell lines with mutated *RB1* and WT *RB1* in TCGA, DepMap, and COSMIC data sets. (**C**–**E**) Distribution of RB1 protein expression in cell lines with mutated *RB1* and WT *RB1* in TCGA and DepMap data sets. (**F**) qRT-PCR analysis of *RB1* levels in 3 *RB1*-WT LUAD cell lines (A549, H1650, and H1975) and 2 *RB1*-mutant LUAD cell lines (H2228 and H1781) (*n* = 6). *P < 0.05, **P < 0.01 vs. *RB1*-WT cells. (**G**) Western blot analysis of RB1 protein expression in *RB1*-WT and *RB1*-mutant LUAD cell lines (*n* = 5). *P < 0.05, **P < 0.01 vs. *RB1*-WT cells. (**H** and **I**) Distribution of ln(IC_50_) (LN_IC50) and AUC in *RB1*-WT and -mutant lung cancer cell lines treated with PARPis in the GDSC data set. (**J** and **K**) Distribution of ln(IC_50_) in RB1-H and RB1-L breast cancer cell lines treated with PARPis in the GDSC data set. (**L**) Distribution of RD and pCR patients with RB1-H and RB1-L in the GSE164458 data set. (**M**) Expression of *RB1* between RD and pCR patients in the GSE164458 data set. *P* values were calculated by 1-sided Wilcoxon’s rank-sum test (**A**–**E**, **H**–**K**, and **M**), unpaired, 2-tailed Student’s *t* test (**F** and **G**), and hypergeometric test (**L**).

LUAD samples, lung squamous cell carcinoma (LUSC) samples, and lung cancer cell lines with *RB1* mutations showed significantly lower expression of *RB1* and encoded proteins than cell lines with WT *RB1* (Figure 3, A–E). In addition, *RB1* expression in *RB1*-mutant cell lines (H2228 and H1781) was lower compared with *RB1*-WT cell lines (A549, H1650, and H1975) (Figure 2A). *RB1* expression analysis was carried out on the LUAD cell lines H2228, H1781, A549, H1650, and H1975 using quantitative real-time PCR (qRT-PCR) and Western blot assay. The results showed that mRNA and protein expression levels of RB1 were downregulated in *RB1*-mutant samples compared with *RB1*-WT samples (Figure 3, F and G). The above analysis indicates that *RB1* mutations are loss-of-function mutations.

We explored the hypothesis that *RB1* expression mediates PARPi sensitivity. Lung cancer cell lines with low *RB1* expression (RB1-L) were sensitive to olaparib according to the pharmacogenomic data in the GDSC data set (Figure 3, H and I). We found that *RB1*-mutant breast cancer cell lines were significantly sensitive to rucaparib and olaparib in the GDSC data set (Figure 3, J and K). Then, TNBC patients treated with veliparib were grouped according to *RB1* expression. There were more patients with pathological complete response (pCR) in the RB1-L group than in the high-*RB1*-expression (RB1-H) group (Figure 3L). In addition, the expression of *RB1* in pCR patients was significantly lower than that in residual disease (RD) patients (Figure 3M). Altogether, our results show that *RB1* mutation is associated with RB1-L and mediates sensitivity to PARPis in lung cancer and breast cancer.

### RB1 deficiency facilitates the effect of PARPis on DNA damage.

**Figure 4:**
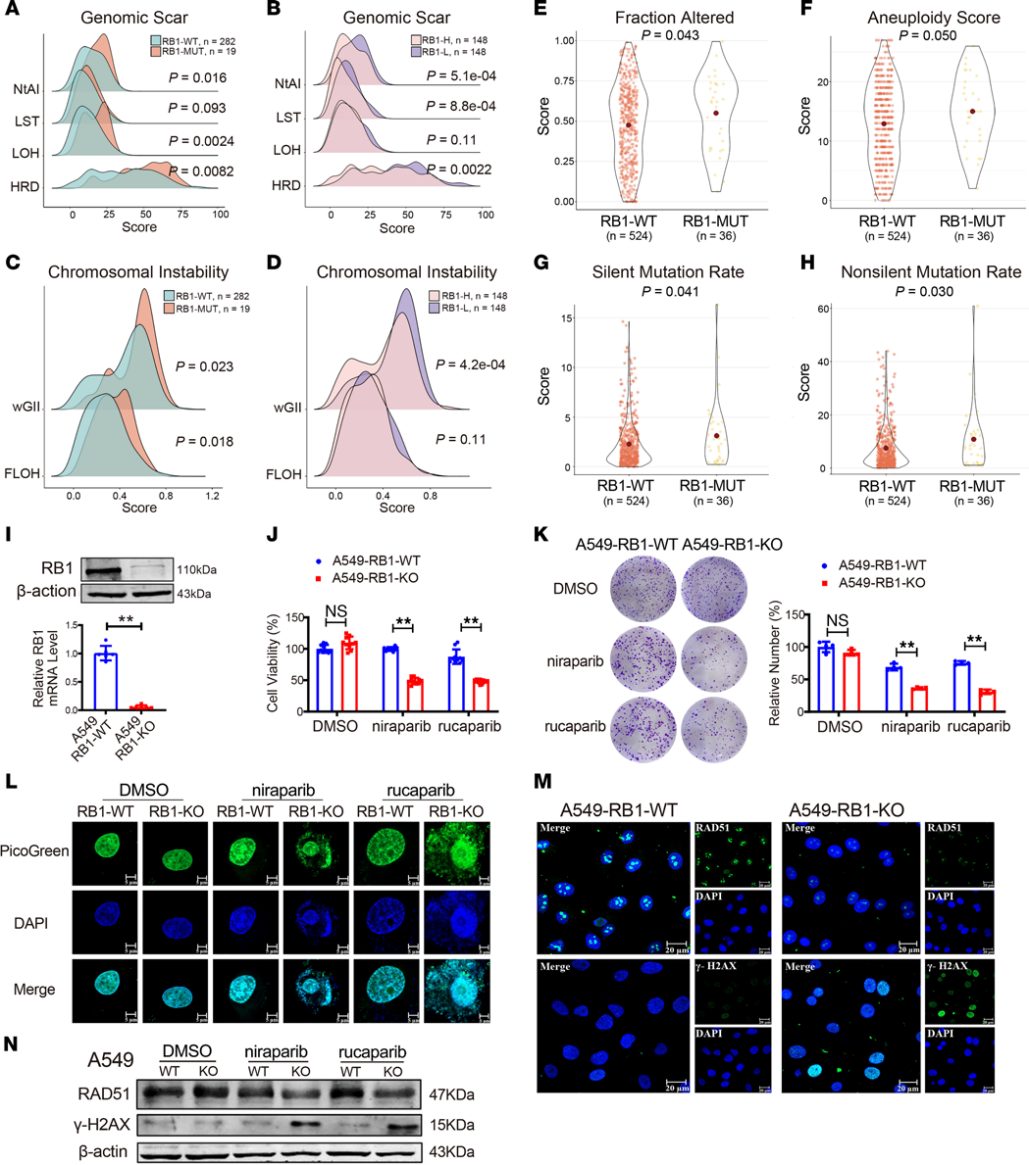
*RB1* deficiency enhances genomic instability. (**A**) Distribution of genomic scar scores between *RB1*-mutant and *RB1*-WT samples. (**B**) Distribution of genomic scar scores between RB1-H and RB1-L samples. (**C**) Comparison of chromosomal instability in *RB1-*mutant and *RB1*-WT samples. (**D**) Comparison of chromosomal instability in RB1-H and RB1-L samples. (**E**–**H**) Comparison of “fraction altered,” “aneuploidy,” “silent mutation rate,” and “nonsilent mutation rate” values between *RB1*-mutant and *RB1*-WT samples. (**I**) Protein and mRNA expression of RB1 in A549-RB1-WT cells and A549-RB1-KO cells assessed by Western blotting and qRT-PCR assays (*n* = 6). **P < 0.01 vs. A549-RB1-WT. (**J**) CCK8 analysis of cell viability (*n* = 10). **P < 0.01 vs. A549-RB1-WT. (**K**) Colony formation assay of A549-RB1-WT and A549-RB1-KO cells treated with 1 μM niraparib, rucaparib, and DMSO for 72 hours followed by 14-day recovery (*n* = 4). **P < 0.01 vs. A549-RB1-WT. (**L**) Representative immunofluorescence images of A549-RB1-WT and A549-RB1-KO cells treated with niraparib, rucaparib, and DMSO for 72 hours. Release of dsDNA into the cytoplasm detected by PicoGreen assay. Green, PicoGreen; Blue, DAPI. Scale bars: 5 μm. (**M**) Immunofluorescence of RAD51 and γ-H2AX in A549-RB1-WT and A549-RB1-KO cells. Scale bars: 20 μm. (**N**) Western blot analysis for RAD51 and γ-H2AX in A549-RB1-WT and A549-RB1-KO cells. *P* values were calculated by 1-sided Wilcoxon’s rank-sum test (**A**–**H**) and unpaired, 2-tailed Student’s *t* test (**I**–**K**).

Some evidence demonstrated that *RB1* mutation increases DNA damage and instability in cancer cells (17). Retinoblastoma localizes to DNA double-strand breaks (DSBs) and promotes DSB repair through HR (18). Next, we explored the relevance between the DNA damage response (DDR) and *RB1* in the TCGA LUAD cohort. *RB1*-mutant samples exhibited significantly higher telomeric allelic imbalances (NtAI), loss of heterozygosity (LOH), and homologous recombination damage (HRD) scores in comparison with WT *RB1* (Figure 4A). Moreover, NtAI, large-scale transition (LST), and HRD scores in RB1-L samples were significantly greater than in RB1-H samples (Figure 4B). Then, we assessed the disparity of chromosomal instability using the weighted genome integrity index (wGII) and the frequency of LOH (FLOH). The wGII and FLOH scores in RB1-mutant samples were significantly higher than RB1-WT samples (Figure 4C), and the wGII scores in RB1-L samples were significantly higher compared with RB1-H samples (Figure 4D). Furthermore, *RB1* mutation was related to increases in “fraction altered,” “aneuploidy,” “silent mutation rate,” and “nonsilent mutation rate” scores (Figure 4, E–H). These results revealed that *RB1* deficiency enhances DDR and induces genomic instability.

As the functions of PARPis are to interfere with DNA repair and induce cell death in cancer cells, we posited that *RB1* might affect cancer cell responses to PARPis by disrupting DNA damage repair. To test this hypothesis, we generated an RB1-KO cell line from *RB1*-WT A549 lung cancer cells using the CRISPR/Cas9 system. *RB1* KO was confirmed with qRT-PCR and Western blotting (Figure 4I). When the same concentration of niraparib and rucaparib was added to cell lines, A549-RB1-KO cell viability was significantly reduced (Figure 4J). We also observed a similar result in cell colony formation assays where the number of cell clones was reduced in A549-RB1-KO cells (Figure 4K). We then examined cytosolic double-stranded DNA (dsDNA) in A549-RB1-WT and A549-RB1-KO cells treated with niraparib and rucaparib using immunofluorescence. As shown in Figure 4L, niraparib and rucaparib promoted the accumulation of cytosolic dsDNA in A549-RB1-KO cells compared with the cells treated with DMSO. More importantly, PARPis did not affect nuclear DNA DSBs in A549-RB1-WT cells, supporting the idea that the presence of *RB1* may partially repair the DNA damage induced by PARPis. Consistently, immunofluorescence analysis of RAD51 (which has a critical role in the repair of DNA DSBs) and γ-H2AX (a key marker of DNA damage) in A549 cells exposed to the same concentration of niraparib indicated that PARPis aggravated the DNA damage response in A549-RB1-KO cells, but not in A549-RB1-WT cells (Figure 4, M and N).

**Figure 5:**
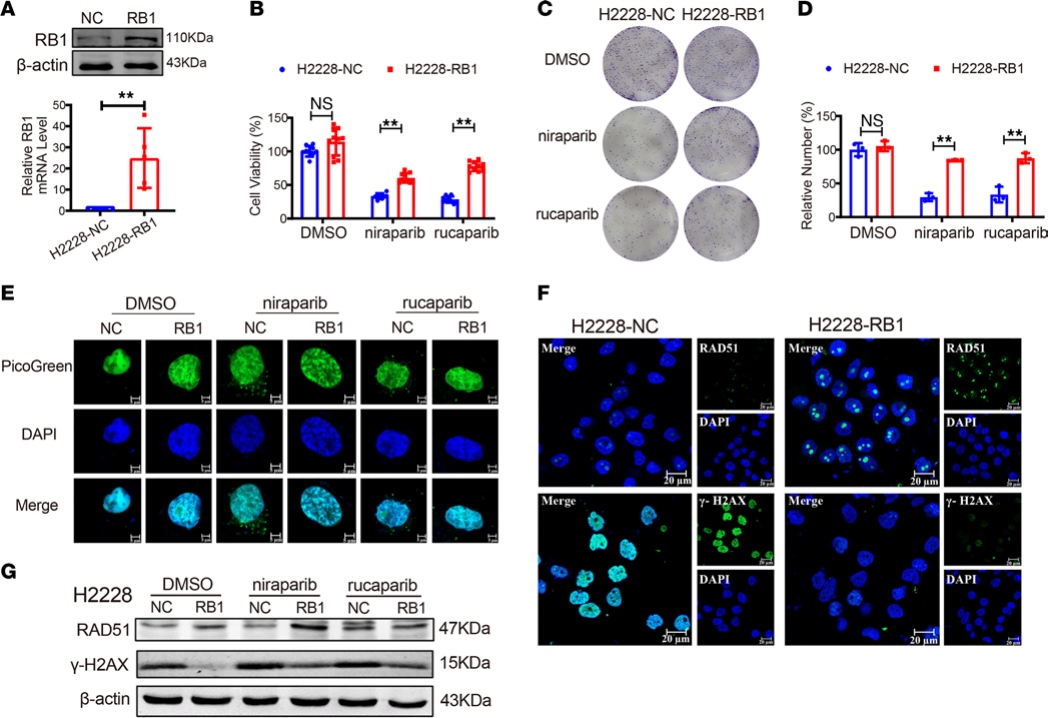
*RB1* overexpression increases DNA damage repair. (**A**) Protein and mRNA expression of *RB1* in H2228 cells transfected with RB1 plasmid assessed by Western blotting and qRT-PCR assays (*n* = 5). **P < 0.01 vs. H2228-NC. (**B**) CCK8 analysis of cell viability after treatment with DMSO, niraparib, and rucaparib (*n* = 10). **P < 0.01 vs. H2228-NC. (**C** and **D**) Representative images (left) and statistical analysis (right) of the colony formation assay in H2228 cells with PARPi treatment (*n* = 3). **P < 0.01 vs. H2228-NC. (**E**) PicoGreen assay detects the release of dsDNA into the cytoplasm after treatment with PARPis in H2228 cells transfected with *RB1*. Green, PicoGreen; Blue, DAPI. Scale bars: 5 μm. (**F**) Immunofluorescence of RAD51 and γ-H2AX. Scale bars: 20 μm. (**G**) Western blot analysis for RAD51 and γ-H2AX in H2228 cells. *P* values in **A**, **B**, and **D** were calculated by unpaired, 2-tailed Student’s *t* test.

To further examine PARPis’ SL interactions with *RB1* in DNA damage repair, we constructed an *RB1* overexpression plasmid and transfected it into *RB1*-mutant cell line H2228 (H2228-RB1). The transfection efficiency was verified by qRT-PCR and Western blotting (Figure 5A). Cell viability assays indicated that overexpression of *RB1* partially restored the proliferation of H2228 cells when given the same dose of niraparib and rucaparib (Figure 5B). We obtained similar results using cell colony formation experiments (Figure 5, C and D). Moreover, we found that niraparib and rucaparib treatments were able to induce the formation of cytosolic dsDNA in H2228, while reexpression of *RB1* protected against DNA damage and reduced the amount of cytosolic dsDNA (Figure 5E). Consistent with this finding, *RB1* reexpression increased the fluorescence intensity of RAD51 and decreased γ-H2AX expression compared with the solvent control group treated with DMSO (Figure 5, F and G). Taken together, our results show that PARPi-mediated activation of DDR was associated with *RB1* gene stabilization in LUAD cells.

### PARPis induce the secretion of chemokines CCL5 and CXCL10 via the cGAS/STING pathway in an RB1-dependent manner.

**Figure 6:**
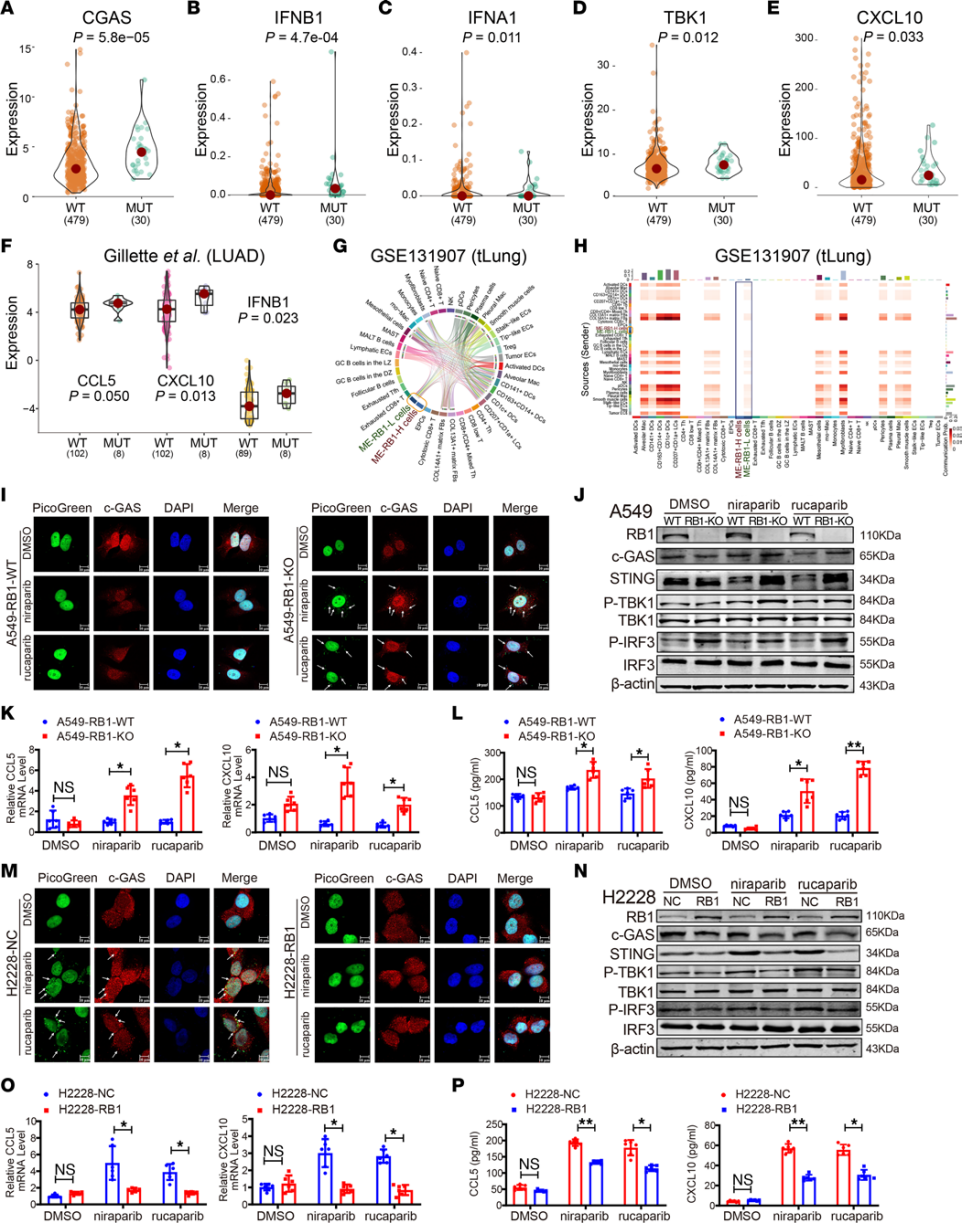
*RB1* deficiency activates innate immune signaling via the cGAS/STING pathway. (**A**–**E**) The comparison of 5 cGAS/STING pathway genes’ expression in TCGA LUAD samples with mutated *RB1* and WT *RB1*. (**F**) Differential expression of 3 cGAS/STING genes between *RB1*-mutant and *RB1*-WT samples in the Gillette et al. data set (19). (**G**) Communications of the GAS signaling pathway among ME-RB1-H, ME-RB1-L, and other cell subtypes. (**H**) Heatmap shows the communicational probability of GAS signaling pathway among cells. The color indicates the strength of communication. (**I**) Representative immunofluorescence images of colocalization of cGAS and cytoplasmic dsDNA fragments induced by PARPis in A549-RB1-WT and A549-RB1-KO cells. (**J**) Western blot analysis of RB1, cGAS, STING, p-TBK1/TBK1, and p-IRF3/IRF3 after treatment with DMSO, niraparib, and rucaparib in A549-RB1-WT and A549-RB1-KO cells. Normalized to β-actin control. (**K**) qRT-PCR analysis of *CCL5* and *CXCL10* mRNAs (*n* = 6). *P < 0.05 vs. A549-RB1-WT. (**L**) Quantitative analysis by ELISA of CCL5 and CXCL10 secretion in A549-RB1-WT and A549-RB1-KO cell supernatants upon exposure to PARPis (*n* = 6). *P < 0.05, **P < 0.01 vs. A549-RB1-WT. (**M**) Representative immunofluorescence images of cGAS and cytoplasmic dsDNA fragment localization induced by PARPis in H2228 cells transfected with *RB1*. (**N**) Western blot analysis of RB1, cGAS, STING, p-TBK1/TBK1, and p-IRF3/IRF3 after treatment with DMSO, niraparib, and rucaparib in H2228 cells transfected with *RB1*. Normalized to β-actin control. (**O**) qRT-PCR analysis of *CCL5* and *CXCL10* mRNAs (*n* = 6). *P < 0.05 vs. H2228-NC. (**P**) Quantitative analysis by ELISA of CCL5 and CXCL10 secretion in H2228 cell supernatants upon exposure to PARPis (*n* = 6). *P < 0.05, **P < 0.01 vs. H2228-NC. (**I** and **M**) Green, PicoGreen. Red, cGAS. Blue, DAPI. The white arrow represents the colocation of cGAS and dsDNA fragments. Scale bars: 10 μm. *P* values were calculated by 1-sided Wilcoxon’s rank-sum test (**A**–**F**) and unpaired, 2-tailed Student’s *t* test (**K**, **L**, **O**, and **P**).

PARPi treatment is known to produce cytosolic dsDNA that is subsequently recognized by cyclic GMP-AMP synthase (cGAS), which leads to the activation of innate immune signaling. In our experiments, *RB1*-mutant samples showed significantly increased expression of several cGAS/STING-related genes in comparison with *RB1*-WT samples in the TCGA LUAD cohort (Figure 6, A–E); *IFNA6*, *IFNA2*, *IFNA14*, and *IFNA8* showed the same trend (Supplemental Figure 2, A–D). In the LUAD cohort of Gillette et al. (19), the expression of *CCL5*, *CXCL10*, and *IFNB1* in *RB1*-mutant samples was significantly higher than in *RB1*-WT samples (Figure 6F). Thus, *RB1* mutation stimulated cGAS/STING signaling.

We further investigated the above findings at single-cell resolution. Cells from 11 early-stage LUAD patients (tLung) in the NCBI Gene Expression Omnibus (GEO) database (GSE131907) were integrated. Cells were annotated into 8 cell types (B lymphocytes, endothelial cells [ECs], epithelial cells, fibroblasts, mast cells, myeloid cells, natural killer [NK] cells, and T lymphocytes) and further divided into cell subtypes. The copy number karyotyping of aneuploid tumors (CopyKAT) algorithm was used to identify malignant cells in the epithelial cell cluster, and further classified by *RB1* expression (see Methods). We applied the CellChat algorithm to infer the communications in GAS signaling among high-*RB1*-expressing malignant epithelial cells (ME-RB1-H), low-*RB1*-expressing malignant epithelial cells (ME-RB1-L), and other cell subtypes. ME-RB1-L cells communicated with multiple cell subtypes via the GAS signaling pathway, including “activated DCs” (dendritic cells) and several subtypes of ECs and fibroblasts (Figure 6G), whereas ME-RB1-H cells did not have communications related to GAS signaling with other cells. The communicational probability of GAS signaling of ME-RB1-L cells with other cells was higher than that of ME-RB1-H cells (Figure 6H).

Then, we investigated whether PARPi-induced cytosolic dsDNA could activate innate immune signaling through the cGAS/STING pathway in *RB1*-mutant LUAD cells. Therefore, we assessed the colocalization of cGAS and dsDNA. Immunofluorescent staining of cGAS and staining with PicoGreen probe in A549-RB1-WT and A549-RB1-KO cell lines exposed to the same concentrations of niraparib and rucaparib revealed a marked increase in the number of cytoplasmic cGAS foci and colocalization with cytosolic dsDNA in A549-RB1-KO cells, but not in A549-RB1-WT cells (Figure 6I). We examined whether cytosolic dsDNA and cGAS could activate STING signaling. Immunoblotting analysis of cGAS, STING, phosphorylation of TBK1 (pSer172 TBK1) and IRF3 (pSer386 IRF3), critical components in the cGAS/STING pathway, were detected in A549 cell lines exposed to PARPis (20). As shown in Figure 6J, niraparib and rucaparib treatment increased cGAS and STING expression in A549-RB1-KO cells, but not A549-RB1-WT cells. Indeed, niraparib and rucaparib treatment also elevated phosphorylation levels of both TBK1 and IRF3 in A549-RB1-KO cells. We then evaluated the mRNA expression level of *CCL5* and *CXCL10*, key downstream targets of STING activation (21). We found that RB1-KO significantly upregulated the mRNA levels of *CCL5* and *CXCL10* induced by PARPis (Figure 6K). Consistent with these changes in mRNA levels, PARPis substantially increased the production of proinflammatory cytokines CCL5 and CXCL10 in A549 cell culture supernatant, as detected by ELISA (Figure 6L).

To investigate whether reexpression of *RB1* restored PARPi-induced DNA DSBs, we overexpressed *RB1* in H2228 cells following PARPi treatment. Analysis of cGAS staining intensity within dsDNA indicated a marked decrease in response to PARPis niraparib and rucaparib in H2228 cells transfected with *RB1* (Figure 6M). Furthermore, we found that overexpression of *RB1* led to the inactivation of components of the STING pathway, including cGAS, STING, p-TBK1, and p-IRF3 in H2228 cells treated with PARPis (Figure 6N). The mRNA levels of *CCL5* and *CXCL10* were downregulated in H2228 cells transfected with *RB1* (Figure 6O). Consistent with these observations, the concentration of CCL5 and CXCL10 in supernatants of H2228-RB1 cells revealed a similar decrease after exposure to PARPis (Figure 6P).

### PARPi suppressed tumorigenesis in RB1-KO mice.

**Figure 7:**
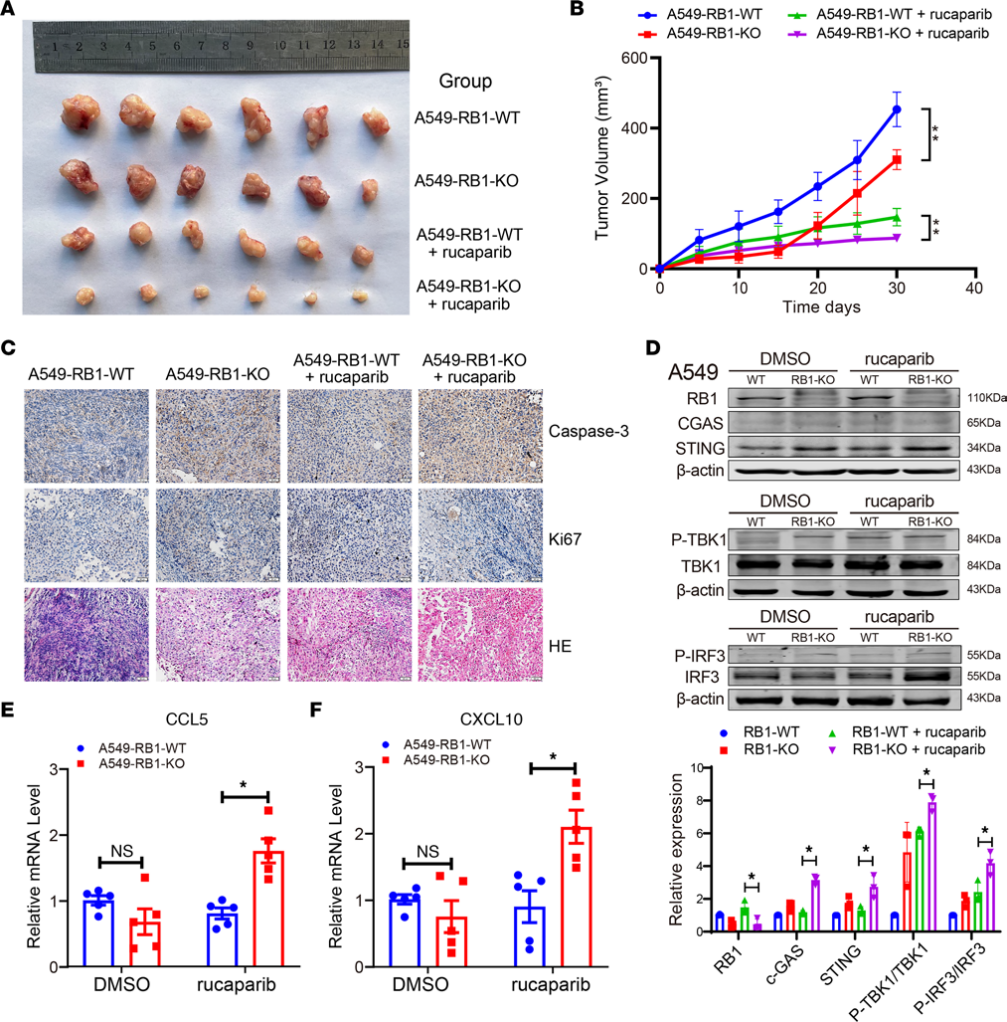
*RB1* KO enhanced the therapeutic efficacy of PARPi in LUAD xenograft. (**A**) Tumor volumes of 4 treatment groups with A549-RB1-WT (100 μL PBS), A549-RB1-KO (100 μL PBS), A549-RB1-WT + rucaparib (60 μL DMSO, 240 μL PEG 300, 30 μL Tween 80, 270 μL normal saline, 1.08 mg rucaparib every 2 days) and A549-RB1-KO + rucaparib (60 μL DMSO, 240 μL PEG 300, 30 μL Tween 80, 270 μL normal saline, 1.08 mg rucaparib every 2 days). (**B**) The tumor growth curves of 4 treatment groups for 30 days. **P < 0.01. (**C**) Representative images of IHC staining in LUAD xenograft samples. Scale bars: 50 μm. (**D**) Western blot analysis of proteins in the STING pathway in LUAD xenograft samples (*n* = 3). (**E** and **F**) qRT-PCR analysis of *CCL5* and *CXCL10* mRNAs in A549-RB1-WT and A549-RB1-KO cells after treatment with PARPis in mice (*n* = 5). *P < 0.05 vs. A549-RB1-WT + rucaparib. *P* values in **D**–**F** were calculated by unpaired, 2-tailed Student’s *t* test.

The above experiments in vitro indicated that *RB1* mutation increased the therapeutic efficacy of PARPis in LUAD cells. Next, to validate the suppressive effect of a PARPi on LUAD in vivo, we established xenograft models in human peripheral blood mononuclear cell–engrafted mice (huPBMC-NOG) to generate human mature T cells. The volume and weight of tumors in mice with A549-RB1-KO cells were significantly reduced compared with A549-WT cells after concurrent treatment with rucaparib (Figure 7, A and B). Immunohistochemical staining showed that the expression of caspase-3 was obviously increased in A549-RB1-KO cells treated with rucaparib. A Ki67 proliferation assay also showed that knockdown of *RB1* obviously increased the antitumor effect of rucaparib (Figure 7C). Western blot analysis revealed that A549-RB1-KO mice treated with rucaparib could activate the cGAS/STING signaling pathway (Figure 7D). qRT-PCR experiments further showed that knockdown of *RB1* increased mRNA expression of the inflammatory factors CCL5 and CXCL10, illustrating the consequent activation of a T cell immune response (Figure 7, E and F).

### RB1 deficiency activates immune infiltration.

**Figure 8:**
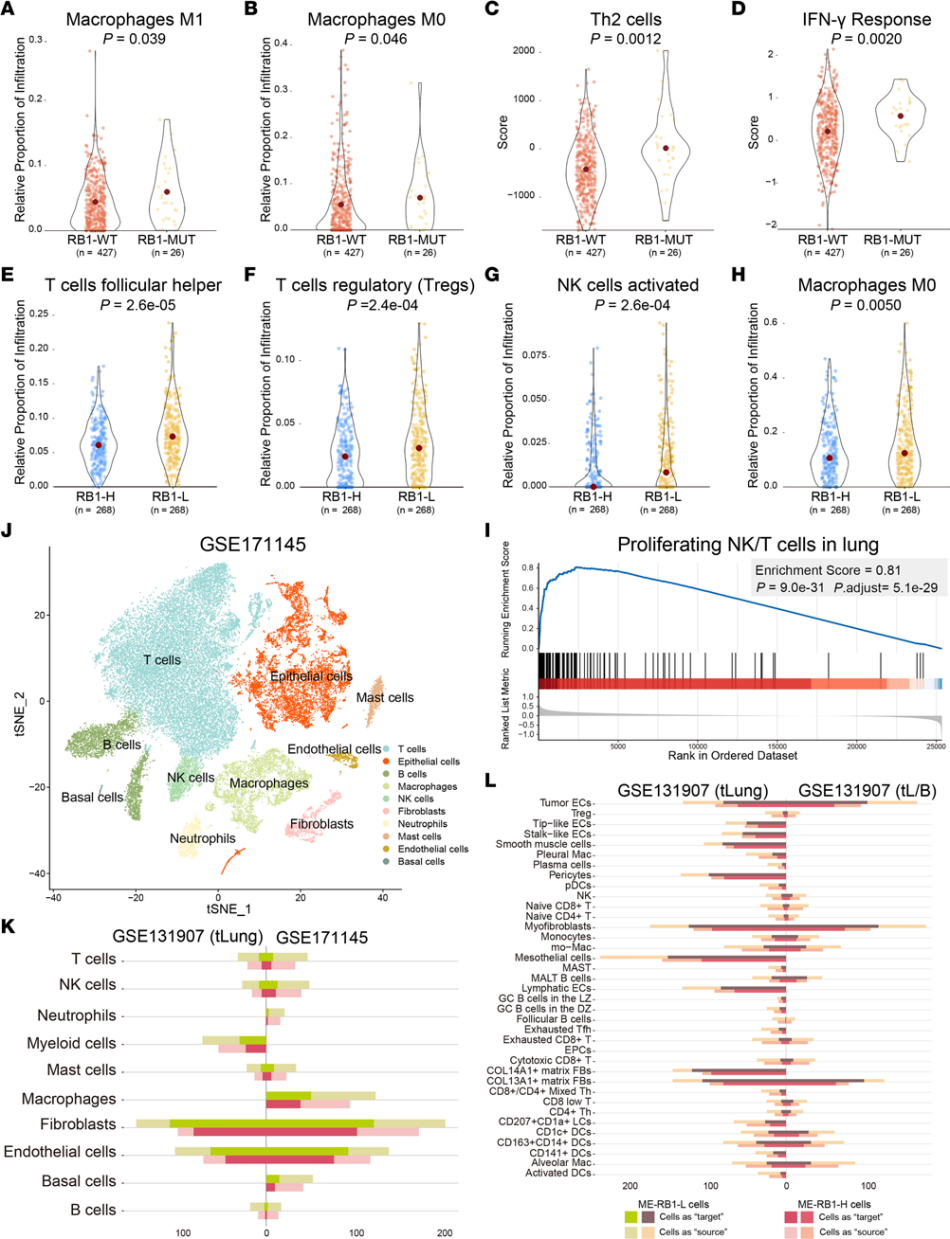
*RB1* deficiency increases immune cell infiltration. (**A**–**C**) Comparison of the “macrophages M1,” “macrophages M0,” and “Th2 cells” infiltration in *RB1*-mutant samples with *RB1*-WT samples. (**D**) Estimation of IFN-γ response between *RB1*-mutant and *RB1*-WT LUAD samples in TCGA. (**E**–**H**) Differential infiltration of “T cells follicular helper,” “T cells regulatory (Tregs),” “NK cells activated,” and “macrophages M0” between RB1-H and RB1-L samples. (**I**) The “proliferating NK/T cells in lung” gene set was significantly enriched in *RB1*-mutant samples. (**J**) The t-distributed stochastic neighbor embedding (tSNE) visualization of LUAD cells in GSE171145, colored by cell types. (**K**) The number of communications between ME-RB1-L or ME-RB1-H with other cell types in the GSE131907 (tLung) and GSE171145 data sets. Green and pink bars indicate communications of ME-RB1-L cells and ME-RB1-H cells, respectively. (**L**) The number of communications between ME-RB1-L or ME-RB1-H with other cell subtypes in early-stage (tLung) and advanced-stage (tL/B) LUAD patients from the GSE131907 data set. Brown and pink bars indicate communications of ME-RB1-L cells and ME-RB1-H cells, respectively. *P* values were calculated by 1-sided Wilcoxon’s rank-sum test (**A**–**H**) and permutation test according to gene set variation analysis (**I**).

Compared with *RB1*-WT samples, fractions of “macrophages M1” (*P* = 0.039) and “macrophages M0” (*P* = 0.046) were significantly higher in *RB1*-mutant samples (Figure 8, A and B). Additionally, “Th2 cells” (T helper 2 cells) (*P* = 0.0012) and “IFN-γ response” (*P* = 0.0020) scores in *RB1*-mutant samples were significantly higher than *RB1*-WT LUAD samples (Figure 8, C and D). Next, we further determined the infiltration distinction between RB1-L and RB1-H samples. Infiltration of “T cells follicular helper” (*P* = 2.6 × 10^–5^), “T cells regulatory (Tregs)” (*P* = 2.4 × 10^–4^), “NK cells activated” (*P* = 2.6 × 10^–4^), and “macrophages M0” (*P* = 0.050) in RB1-L samples was higher than in RB1-H samples (Figure 8, E–H). The “proliferating NK/T cells in lung” gene set was significantly enriched in *RB1*-mutant samples (Figure 8I).

To gain insight into the discrepancy in the immune microenvironment between RB1-L and RB1-H samples, we performed cell-cell communication analysis at the single-cell level. After eliminating the batch effect by using the R package “harmony,” cells collected from 9 LUAD samples in GSE171145 were merged and clustered, in which samples showed uniform distribution (Supplemental Figure 3 and Supplemental Figure 4A). We annotated clusters by the singleR method and expression of canonical marker genes (Supplemental Figure 4, B and C, and Supplemental Figure 5A). A total of 10 distinct cell clusters were identified, including T cells, epithelial cells, B cells, macrophages, NK cells, fibroblasts, neutrophils, mast cells, ECs, and basal cells (Figure 8J).

In the GSE131907 (tLung) and GSE171145 data sets, the number and strength of interactions between ME-RB1-L cells and other cell types were higher than those between ME-RB1-H cells and other cell types, both in sender and receiver cells (Figure 8K, Supplemental Figure 5B, and Supplemental Figure 6A), especially tissue stem cells and macrophages in GSE171145, fibroblasts and myeloid cells in GSE131907 (tLung), ECs, T cells, and NK cells in both data sets. Similarly, subtype cells frequently communicated with ME-RB1-L cells compared with ME-RB1-H in both early-stage and advanced-stage LUAD patients in GSE131907 (Figure 8L), and the level of communication strengthened (Supplemental Figure 6B). In addition, we investigated specific communications in ME-RB1-L cells. In the GSE171145 and GSE131907 (tLung) data sets, interactions of secreted phosphoprotein 1 (SPP1), laminin, intercellular adhesion molecule (ICAM), and major histocompatibility complex class II (MHC-II) signaling pathway were specifically present in ME-RB1-L cells, indicating immune upregulation in these cells (Supplemental Figure 7, A and B).

## Discussion

PARPis target *PARP* genes by reducing DNA repair function and increasing replication fork errors in cancer cells. *RB1* is located in the nucleoplasm (Supplemental Figure 8) and performs DDR functions. We revealed that loss-of-function mutations of *RB1* induce DNA damage and promote dsDNA accumulation in the cytoplasm. DSBs are subsequently recognized by the cGAS/STING pathway, followed by triggering of innate immune signaling. Immune cells (e.g., macrophages, T cells, NK cells, DCs, and B cells) are recruited to the surface of lung cancer cells and destroy tumor cells (Supplemental Figure 7C). Our study indicated that the immunomodulatory capacity of PARPis could improve PARPis’ efficacy in *RB1*-mutant LUAD patients.

Some studies showed that increasing *PARP1* expression dictated the sensitivity to PARPis (22). Our study revealed that the expression of *PARP1* and *PARP2* was significantly upregulated in *RB1*-mutant samples. Moreover, we found a negative correlation between *PARP1/2* expression and *RB1* expression, which also confirmed our assumption that *RB1* deficiency mediates sensitivity to PARPis based on SL interactions for LUAD.

PARPis have the potential to treat multiple cancers. In 2023, the US Food and Drug Administration approved talazoparib with enzalutamide for metastatic castration-resistant prostate cancer patients with mutations of an HR repair gene (*ATM*, *ATR*, *BRCA1*, *BRCA2*, *CDK12*, *CHEK2*, *FANCA*, *MLH1*, *MRE11A*, *NBN*, *PALB2*, or *RAD51C*), and approved niraparib for advanced ovarian, fallopian tube, or primary peritoneal cancer patients with HRD-positive status in 2019 (23, 24). Moreover, in phase Ib and II trials of avelumab plus talazoparib treatment in NSCLC patients, the overall response rate of DDR-positive patients was higher than DDR-WT patients (25). In this study, our results suggest that LUAD patients with *RB1* mutation may benefit from PARPis due to the increase in DNA damage and immune infiltration. Currently, there is a lack of publicly available PARPi-response data for LUAD patients with corresponding transcriptome or genome sequencing. *RB1* is an important regulator of the cell cycle, and functional inactivation of *RB1* has been recognized to occur in multiple human cancers, including TNBC (26). TNBC and LUAD have common oncogenetic mechanisms (27, 28); therefore, PARPi-response data for TNBC was used to verify the therapeutic effect of *RB1* on PARPis (Figure 3, J–M). Moreover, *RB1* mutation has been verified to induce PARPi hypersensitivity in osteosarcoma tumor cells (29). Hence, development of basket clinical trials to access the efficacy and safety of PARPis in *RB1*-mutant LUAD patients is a priority in the near future.

In this study, we overexpressed *RB1* using a constructed plasmid (pcDNA3.1 vector, 0.2 μg) and found increased fluorescence intensity of RAD51 and decreased γ-H2AX expression, indicating that overexpressed *RB1* may increase the capacity of DNA damage repair (Figure 5, F and G). Tang et al. transfected a gastric cancer cell line with 2 concentrations of pcDNA3.1 vector (0.2 μg and 0.5 μg) to overexpress *RB1* and derived consistent results (30). In this study, whether the effect of *RB1* is dose dependent in lung cancer cells warrants future detailed research.

Previous studies have reported that PARPis induce innate immunity via PARP1 trapping–induced DNA damage and activation of cGAS/STING signaling (31). Our study indicated a synergistic effect of *RB1* mutation and treatment with PARPis on the activation of cGAS/STING. Our findings indicated that *RB1* deficiency significantly upregulated the mRNA and protein levels of CCL5 and CXCL10 induced by PARPis. According to CellChat analysis, CCL signaling specifically communicated in ME-RB1-L cells, and CCL5 sends communications to atypical chemokine receptor 1 (ACKR1) and C-C chemokine receptor type 1 (CCR1) (Supplemental Figure 7A). CCL5 and its receptors are critical for the recruitment of effector immune cells to the site of inflammation. *CXCL10* and *CCL5* overexpression is associated with the presence of CD8^+^ T cells in lung cancer (21). Taken together, the results indicate the crucial role of CCL5 and CXCL10 in mediating T cell recruitment in *RB1*-deficient cancer cells.

We revealed that type I IFNs were significantly upregulated in *RB1*-mutant LUAD samples (Figure 6, B and C, and Supplemental Figure 2). The type II IFN (IFN-II), IFN-γ, plays an important role in both innate and adaptive immunity, which was upregulated in *RB1*-mutant samples (32). IFN-II and MHC-II signaling pathways specifically communicate in ME-RB1-L cells (Supplemental Figure 7, A and B). IFN-II is primarily secreted by adaptive immune cells, and upregulates MHC-II expression on antigen-presenting cells. Previous studies revealed that PARPis upregulate MHC-II on DCs, enhancing antigen presentation and T cell interactions (33). In addition, numerous regulators of SPP1 and ICAM appeared to have specific interactions in ME-RB1-L cells (Supplemental Figure 7, A and B). SPP1 is a cytokine that upregulates the expression of IFN-γ, and is related to cytokine activity and extracellular matrix binding. ICAM-1 is a cell adhesion molecule that plays a key role in cell-cell interactions leading to the immune response. Our analysis demonstrated the upregulation of cytokine activity and cell adhesion–activated immune response in *RB1*-deficient LUAD samples, and an increase in adaptive and innate immunities. Moreover, the intercellular communications were inferred by CellChat based on single-cell RNA-seq (scRNA-seq) data; the accuracy of the interactions among cells need further experimental validation.

Infiltration of immune cells was evaluated in LUAD bulk tissue. M0/M1 macrophages, T cells, and activated NK cells were upregulated in *RB1*-deficient cells. Furthermore, the communications between ME-RB1-L cells and immune cells was higher compared with ME-RB1-H cells at single-cell resolution. Taken together, these results prompted us to conjecture that the immune cells (e.g., macrophages, T cells, NK cells, DCs, and B cells) are recruited to the surface of *RB1*-mutant lung cancer cells (Supplemental Figure 7C). A deeper understanding of the change in the TME after PARPi treatment in *RB1*-mutant LUAD patients warrants further investigation.

In summary, our study demonstrated the remarkable efficacy of PARPis in treating *RB1*-mutant LUAD cells, which provides a rationale for the therapy in clinical settings and a new treatment strategy for LUAD.

## Methods

### Multiomics data of lung cancer cell lines and bulk LUAD samples.

We downloaded gene mutation and expression data of 182 lung cancer cell lines from the Catalogue of Somatic Mutations in Cancer (COSMIC v87, https://cancer.sanger.ac.uk/cosmic; accessed December, 2018), and obtained somatic mutation, gene expression, and protein expression data for 244 lung cancer cell lines from the DepMap portal v19Q1 (https://depmap.org/portal/; accessed March, 2019). RNA interference (RNAi) screen data were downloaded from the DepMap Portal, Achilles v2.20.2.

Somatic mutation, gene expression, and protein expression data of LUAD and LUSC patients from TCGA were downloaded from the Genomic Data Commons (https://portal.gdc.cancer.gov/; accessed March, 2019). Gene expression and *RB1*-mutant information of 110 LUAD patients was obtained from the study of Gillette et al. (19). Silent mutations and mutations in 5′-flanks, introns, intergenic regions, 5′-UTR, and 3′-UTR were excluded (13).

### Pharmacological data of PARPis.

Pharmacological screening data of PARPis in lung cancer cell lines were obtained from the GDSC v17Jul19 (https://www.cancerrxgene.org/; accessed September, 2019) and the CTRP v2.1 (https://portals.broadinstitute.org/ctrp.v2.1/; accessed January, 2018). We obtained ln(IC_50_) (natural log of the fitted half-maximal inhibitory concentration) values and AUC (area under the drug inhibition curve) values of PARPis. Cell line information for GDSC was referenced from COSMIC, and cell line information for CTRP was available from the DepMap portal.

Drug response and gene expression data of TNBC patients from a randomized phase III trial, BrighTNess, were downloaded from the GEO (GSE164458). We focused on 237 TNBC patients who were treated with veliparib plus DNA-damaging agents (carboplatin and paclitaxel), 127 TNBC patients were labeled as pCR, and 110 TNBC patients achieved RD. Expression values were log_2_ normalized.

### scRNA-seq data processing and analysis.

The scRNA-seq data of 9 LUAD samples were obtained from GSE171145. We used R package “harmony” (https://cran.r-project.org/web/packages/harmony/index.html) to integrate cells across individuals. scRNA-seq data were clustered and annotated according to the singleR package (https://bioconductor.org/packages/release/bioc/html/SingleR.html) and canonical marker genes. scRNA-seq data of 11 early-stage LUAD patients (tLung) and 4 advanced stage LUAD patients (tL/B) in the study of Kim et al. were obtained from GSE131907 (34). scRNA-seq data were annotated according to canonical marker genes in Kim et al. (Supplemental Table 2).

We performed the CopyKAT algorithm (https://github.com/navinlabcode/copykat) to estimate genomic copy number profiles. Epithelial cells labeled as “aneuploid” were considered as malignant cancer cells. *RB1*-expressing malignant epithelial cells were grouped by median of *RB1* expression into ME-RB1-H and ME-RB1-L. The cell-cell communications were analyzed using the R package “CellChat.” See Supplemental Methods for details.

### Evaluation of chromosomal and genomic instability.

Integrated previously published measures of genomic scar and chromosomal instability of LUAD samples in TCGA were obtained from Marquard et al. (35). Genomic scar signatures include the NtAI, LST, and LOH. The sum of the 3 scores is titled the HRD score. Chromosomal instability measures contain the wGII and the FLOH.

The “aneuploidy” score reports the total number of arm-level amplifications and deletions in cancer samples. The “fraction altered” means somatic copy number alterations divided by copy number variation. The 2 measures of LUAD samples in TCGA were obtained from Knijnenburg et al. (36). In addition, the measures of “nonsilent mutation rate” and “silent mutation rate” for LUAD samples in TCGA were acquired from Thorsson et al. (37).

### Components in cGAS/STING pathway.

A total of 31 genes in the cGAS/STING pathway were collected from the Kyoto Encyclopedia of Genes and Genomes (KEGG, https://www.genome.jp/kegg/) database, and studies by Chen et al. (38), Motwani et al. (39), Chabanon et al. (40), and Loo et al. (41) (Supplemental Table 1).

### Evaluation of immune cell infiltration in bulk LUAD samples.

Immune cel’l fractions of TCGA LUAD samples were estimated by the Cell type Identification By Estimating Relative Subsets Of RNA Transcripts (CIBERSORT) algorithm (https://cibersortx.stanford.edu/cshome.php). The fraction of “Th2 cells” in TCGA LUAD samples was obtained from Thorsson et al. (37). Estimation of “IFN-γ response” in TCGA LUAD samples was acquired from the study of Wolf et al. (42).

Cell type signature gene sets curated from cluster markers identified in scRNA-seq studies of human tissues were downloaded from the Molecular Signatures Database (MSigDB: https://www.gsea-msigdb.org/gsea/msigdb; accessed December, 2021). The R package “limma” was used to identify differentially expressed genes between *RB1*-mutant and *RB1*-WT LUAD samples in TCGA, and Gene Set Enrichment Analysis (GSEA) was used to explore the cell type that significantly differentially infiltrated *RB1*-mutant samples by R package “clusterProfiler.”

### Survival analysis.

See Supplemental Methods for details.

### Cell culture.

The human LUAD cancer cell lines A549, H1650, H1975, H2228, and H1781 in this study were purchased from the Chinese Academy of Sciences. H1650, H1975, H1781, and H2228 were cultured in RPMI 1640 medium (VivaCell) with 10% fetal bovine serum (FBS, VivaCell), 100 U/mL penicillin, and 100 μg/mL streptomycin. A549 were maintained with an F-12K Nutrient Mixture (Gibco). All cells were maintained at 37°C with 5% CO_2_ in a humidified incubator. All cell lines were short tandem repeat (STR) typed to confirm identity before the study.

### CRISPR/Cas9-mediated RB1-KO cell line.

For RB1-KO cells, single guide RNAs (sgRNAs) were designed using the online CRISPR design tool (Red Cotton, https://en.rc-crispr.com/). See Supplemental Methods for details.

### Chemical inhibitors.

The PARPi olaparib (AZD2281) was purchased from Selleck Chemicals. Rucaparib (AG014699) and niraparib (MK4827) were purchased from MedChemExpress. Inhibitor stock solutions were prepared in DMSO and stored in aliquots at –80°C. According to DrugBank (https://go.drugbank.com/), the targets of olaparib and rucaparib are *PARP1*, *PARP2*, and *PARP3*, and the targets of niraparib are *PARP1* and *PARP2*.

### Cell viability measurement.

CCK8 assay was used to assess the cell growth. Approximately 1 × 10^5^ cells/well were plated in a 96-well plate. After treatment, the cells were incubated with CCK8 (TransGen Biotech) solution at 37°C for 2 hours. The absorbance was measured at 450 nm using an Infinite 200PRO microplate spectrophotometer (Tecan).

### Colony formation.

The cells were seeded in a 6-well culture plate. Following drug incubation for 72 hours, the medium was aspirated, and cells were incubated with no-drug media for 14 days. Next, the cells were fixed with cold methanol for 20 minutes, stained with 0.1% crystal violet for 15 minutes, washed with PBS, and imaged. Colonies were counted manually using a stereomicroscope and quantitated using Image-Pro Plus software (Media Cybernetics).

### Xenograft model.

The huPBMC-NOG-dKO models were used to generate human mature T cells. Six- to 8-week old huPBMC-NOG-dKO female mice were purchased from Beijing Vital River Laboratory Animal Technology. Cells (5 × 10^6^) containing A549-RB1-KO or A549-WT were suspended in 100 μL PBS, and A549-WT was transplanted subcutaneously into the mouse’s left armpit and A549-RB1-KO was subcutaneously transplanted into the mouse’s right armpit. The dosing group was injected with 10 mg/kg rucaparib intraperitoneally every 2 days (1.08 mg rucaparib, 60 μL DMSO, 240 μL PEG 300, 30 μL Tween 80, and 270 μL normal saline; 100 μL/time each). Tumor volume was measured every 4 days with vernier calipers. Animals were randomly assigned to experimental groups. During the experiment, the researchers did not know the population distribution when evaluating the results. The sample size for each condition was 6. All mice were sacrificed after 4 weeks.

### IHC analysis.

Samples collected from huPBMC-NOG-dKO female mice tumor tissue sections were dewaxed in xylene and rehydrated with gradient ethanol. After washing with double-distilled water, tissue sections were incubated with 3% hydrogen peroxide (Solarbio) for 10 minutes and antigen retrieval was performed with sodium citrate antigen recovery solution (Solarbio). Then, sections were blocked with 50% goat serum for 1 hour, incubated overnight with anti–caspase-3 (Wanleibio; catalog WL01927; 1:200) and anti-Ki67 (Affinity; catalog AF0198; 1:100) primary antibodies at 4°C, and then secondary antibody conjugated with horseradish peroxidase (HRP) (Zhongshan Science and Technology) for 30 minutes at 37°C. The tissue sections were stained with DAB (Zhongshan Science and Technology) staining solution, and then reverse stained with hematoxylin (LABEST) for 10 seconds. Photographs were obtained using a Zeiss microscope.

### H&E staining.

Tissues were fixed in 4% paraformaldehyde for 24 hours, embedded in paraffin, and cut into 5-μm-thick sections. The sections were then stained with H&E (Solarbio) according to the manufacturer’s instructions and observed under a Zeiss microscope.

### TUNEL staining.

TUNEL assay was performed to detect apoptotic cells using an in situ cell death detection kit (fluorescein, Roche Applied Science) according to the manufacturer’s instructions. See Supplemental Methods for details.

### Western blotting.

See Supplemental Methods for details.

### qRT-PCR.

See Supplemental Methods for details.

### Immunofluorescent staining.

See Supplemental Methods for details.

### PicoGreen staining.

PicoGreen staining was performed using Quant-iT Pico-Green dsDNA reagent kits from Thermo Fisher Scientific. See Supplemental Methods for details.

### ELISA.

Detection of cytokines CCL5 and CXCL10 in cell supernatants was measured by ELISA kits (Proteintech, KE00093 and KE00128, respectively). See Supplemental Methods for details.

### Statistics.

One-sided Wilcoxon’s rank-sum test was used to assess the discrepancy between *RB1*-mutant and -WT samples, or to test the difference between RB1-L and RB1-H samples, which were separated by the median expression value of *RB1*. The correlations between *PARP1/2/3* expression and *RB1* expression were evaluated by Pearson’s correlation analysis. Data are presented as mean ± SD, and the unpaired, 2-tailed Student’s *t* test was used to compare the mean values of independent samples unless otherwise noted. A *P* value of less than 0.05 was considered statistically significant. Statistical analyses were performed using R software v4.1.2 (https://www.r-project.org) and GraphPad Prism software v8.0 (https://www.graphpad.com/scientific-software/prism/).

### Study approval.

All animal procedures were approved by the Animal Experiment Ethics Committee of the Harbin Medical University (no. IRB3022823), Harbin, China.

### Data availability.

The public data used in this study can be downloaded from public databases or retrieved from associated files of papers. All data generated or analyzed during this study are included in the article and its Supporting Data Values file. The underlying data generated as part of this study are available from the corresponding author upon request.

## Author contributions

YG and YH conceptualized the study. QD and TY developed the methodology. QD, TY, BC, ML, XS, HC, and ZJ carried out the investigation. QD, TY, BC, and ML analyzed the data. QD, TY, XS, and HC generated figures. QD, TY, KL, and HX validated the data. QD and TY wrote the original draft of the manuscript. YG, YH, HL, KL, HX, YW, and SZ reviewed and edited the manuscript. The order of co–first authors was assigned according to discussions and votes by all the authors.

## Supplementary Material

Supplemental data

Supporting data values

## Version 1 11/08/2023

Electronic publication
