# Supplementary material for: Mutant RB1 enhances therapeutic efficacy of PARPis in lung adenocarcinoma by triggering the cGAS/STING pathway
Source: JCI Insight. 2023 Nov 8;8(21):e165268. doi: 10.1172/jci.insight.165268 (PMC10721263; doi:10.1172/jci.insight.165268)
Supplement: Supplemental data [file jciinsight-8-165268-s009.pdf]

Mutant *RB1* Enhances the Therapeutic Efficacy of PARPis in Lung Adenocarcinoma by Triggering cGAS-STING Pathway

Supplementary Materials

Supplemental Methods 2

Single-cell RNA sequencing data processing and analysis 2
Survival analysis 3
CRISPR/Cas9-mediated *RB1* knockout cell line 3
TUNEL staining 4
Western blot 4
Quantitative real-time PCR 5
Immunofluorescence staining 6
PicoGreen staining 6
Enzyme-linked immunosorbent assay (ELISA)..6

Supplemental Tables..**8**

Supplemental table 1. Information of cGAS-STING pathway genes 8
Supplemental table 2. Marker genes from the dataset of GSE131907 9

Supplemental Figures..**11**

Supplemental figure 1. Correlation of the expression of *RB1* and *PARP1*...........11
Supplemental figure 2. Differential expression of four type I interferon genes....12
Supplemental figure 3. The clustering tree at multiple resolutions.......................13
Supplemental figure 4. Identification of cell clusters and cell type annotation in GSE171145..14
Supplemental figure 5. Expression of canonical marker genes and communications among cell types in GSE171145..16
Supplemental figure 6. Analysis of communications among cell types in GSE131907.. 17
Supplemental figure 7. Schematic diagram of therapeutic mechanism................ 18
Supplemental figure 8. *RB1* localizes in the nucleoplasm 20

Supplemental Methods

Single-cell RNA sequencing data processing and analysis

The scRNA-seq data of nine samples collected from eight lung adenocarcinoma (LUAD) patients were obtained from GSE171145. The BD Rhapsody system was used to capture the transcriptomic information of the sample-derived single cells. For cell-quality filtering, we removed cells with < 200 expressed genes and a mitochondria UMI rate of > 40%, and mitochondrial genes were removed from the expression table. In order to remove batch effects among samples while preserving biological variation, we used R package "harmony" (v 0.1.0) to integrate cells across individuals. First, we searched highly variable genes using a variance-stabilizing transformation method from the "Seurat" (v 4.1.0) package in R. Top 2000 highly variable genes were selected for principal component analysis (PCA). Then, we ran "Harmony" on the first 30 PCs, and performed clustering by the smart local moving (SLM) algorithm with a resolution of 0.4, obtained the corrected PC embeddings finally. Cells were visualized using a 2-dimensional tSNE on the same distance metric. Cell type initial annotation was performed with "SingleR" (v 1.8.1) package using HumanPrimaryCellAtlasData provide by "celldex" (v 1.4.0) package, then the cell type annotation was adjusted by the expression of canonical cell type marker genes.

Another processed scRNA-seq data with 11 early stage (stage I, stage II, and stage III) LUAD patients (tLung) and four advanced stage (stage IV) LUAD patients (tL/B) were obtained from GSE131907. Cells were subjected to scRNA-seq using 10x Genomics. The raw gene-cell-barcode matrix was performed quality control and lognormalized to TPM-like values (log2 [TPM + 1]). Then, scRNA-seq data were clustered, followed by annotating according to known marker genes using Seurat algorithm.

To detect malignant cancer cells, we performed CopyKAT algorithm by R package "copykat" (v 1.0.8) to estimate genomic copy number profiles using gene

expression profiles. Epithelial cells which labeled as “aneuploid” were considered as malignant cancer cells, and further reserved malignant epithelial cells in which retinoblastoma tumor suppressor gene (*RB1*) expressed (expression value non-zero). *RB1* expressed malignant epithelial cells were grouped by median expression of *RB1* into high expressed cells (ME-RB1-H) and low expressed cells (ME-RB1-L). A total of 769, 436, and 208 ME-RB1-L cells and 769, 435, and 207 ME-RB1-H cells in GSE171145, GSE131907 (tLung), and GSE131907 (tL/B), respectively.

The cell-cell communication among ME-RB1-H, ME-RB1-L and other type cells were analyzed using the R package “CellChat” (v1.4.0). We focused on the human database in CellChat and identified over-expressed ligands or receptors by “identifyOverExpressedGenes” and “identifyOverExpressedInteractions” functions. Then, we mapped gene expression data onto PPI network by “projectData” function. “computeCommunProb” and “filterCommunication” functions (min.cells = 10) were used to compute communication probability and infer cellular communication network. “computeCommunProbPathway” and “aggregateNet” functions were used to infer the cell-cell communication at a signaling pathway level between each cell type. We identified the communications between ME-RB1-L cells and other cell types (excluded ME-RB1-H cells), and the communications did not appear in ME-RB1-H cells simultaneously, defined as specific communications in ME-RB1-L cells.

Survival analysis

Overall survival data were downloaded from TCGA. LUAD patients with *RB1* mutation were grouped according to the median expression of *PARP1*. Log-rank test was used to assess the difference in the survival time between two group patients. Kaplan-Meier plots were used to present the results.

CRISPR/Cas9-mediated *RB1* knockout cell line

For *RB1* knockout (RB1-KO), the single guide RNAs (sgRNAs) were designed using the online CRISPR design tool (Red Cotton^{TM}, Guangzhou, China,

https://en.rc-crispr.com/). The exon 1 region of *RB1* was selected to be targeted by CRISPR/Cas9 genome editing. A ranked list of sgRNAs was generated with specificity and efficiency scores. The pair of oligos for two targeting sites were annealed and ligated to the YKO-RP006 vector (Ubigene Biosciences Co., Ltd., Guangzhou, China). The YKO-RP006-hRB1[gRNA] plasmids containing each target sgRNA sequence were transfected into cells with Lipofectamine 3000 (Thermo Fisher Scientific). 24-48 hours after the transfection, puromycin was added to screen the cells. After antibiotic selection, a certain number of cells were diluted by a limited dilution method and inoculated into a 96-well plate. Selection of single clones was performed after 2-4 weeks and selected RB1-KO clones were validated by qRT-PCR and Sanger sequencing. The gRNA sequences were CTGACGAGAGGCAGGTCCTCCGG.

TUNEL staining

TUNEL assay was performed to detect apoptotic cells using an in situ cell death detection kit (fluorescein, Roche Applied Science) according to the manufacturer's instructions. Cell slides were fixed in 4% paraformaldehyde (Solarbio, Beijing, China) and permeabilized with 0.1% Triton X-100 in PBS. The treatment group was mixed with 50u1 TdT and 450ul labeled dUTP solution, and the reaction was performed for 60 min at 37°C in a humidified atmosphere in the dark. DAPI was prepared to stain nuclei. The number of TUNEL-positive cells (green cells) and the total number of cells (blue cells) were measured by Image-Pro Plus.

Western blot

Cells were lysed in RIPA buffer with a cocktail of phosphatase inhibitor and protease inhibitor. Protein concentration was determined by BCA Protein Assay Kit (Beyotime, Shanghai, China), The protein lysates were mixed with loading buffer and boiled at 100 °C for 7 min. Protein samples were separated on 10% or 12% SDS-PAGE. Then transferred the proteins onto the nitrocellulose membrane. The

membrane was incubated with the corresponding primary antibody, including RB1 (Proteintech, Chicago, USA 1:500, 10048-2-Ig), P-TBK1 (Cell Signaling Technology, Boston, USA 1:500, #5483), TBK1 (Cell Signaling Technology, 1:1000, #38066), P-IRF3 (Cell Signaling Technology, 1:500, #37829), IRF3 (Cell Signaling Technology, 1:1000, #4302), C-GAS (Proteintech, 1:1000, 26416-1-AP), RAD51 (Proteintech, 1:1000, 14961-1-AP), r-H2AX (Abcam, UK, 1:2000, ab2893), STING (Cell Signaling Technology, 1:1000, #13647). The protein bands were analyzed by Odyssey Infrared Imaging System (Odyssey, LICOR, USA).

Quantitative real-time PCR

Total RNA was extracted from cells using TRIzol reagent (Invitrogen, Carlsbad, CA, USA) according to the manufacturer's protocol. The concentration and purity of RNA were detected by NanoDrop 8000 (Thermo, USA) and reverse transcribed to cDNA using All-in-One First-Strand cDNA Synthesis SuperMix (TransGen Biotech, Beijing, China, AT341-02). qRT-PCR was performed by the ABI QS6 system (Applied Biosystems, ABI, USA). The data was analyzed by $2^{-\Delta\Delta Ct}$ and normalized to *Actb* in each sample.

The following primer sequences were used:

RB1-Forward	5'- TCCAGACCCAGAAGCCATTGAAATC -3'
RB1-Reverse	5'- GGTGCTCAGACAGAAGGCGTTC -3'
CCL5-Forward	5'- ATTTGCCTGTTTCTGCTTGCTCTTG -3'
CCL5-Reverse	5'- AACTGCTGCTGTGTGGTAGAATCTG -3'
CXCL10-Forward	5'- CTCTCTCTAGAACTGTACGCTG -3'
CXCL10-Reverse	5'- ATTCAGACATCTCTTCTCACCC -3'
ACTB-Forward	5'- GGGAAATCGTGCGTGACATT -3'
ACTB-Reverse	5'- GGAACCGCTCATTGCCAAT -3'

Immunofluorescence staining

Cells were fixed in 4% paraformaldehyde for 30 minutes at room temperature before staining and permeabilized with 0.4% Triton X-100 in PBS for 30 minutes. After 3 additional washes in phosphate-buffered saline (PBS) for 5 min, cells were blocked with Goat Serum (Boster, California, USA) for one hour at room temperature. Then cells were incubated with primary antibodies RAD51 (Proteintech, 14961-1-AP, 1:200), cGAS (Proteintech, 26416-1-AP, 1:200), and γ-H2AX (Abcam, ab2893, 1:200) at 4°C overnight. The secondary antibodies were incubated with Alexa Fluor 594–conjugated mouse or rabbit and Alexa Fluor 488–conjugated rabbit at 1:200 dilution for 1 hour at room temperature. After being washed with PBS, slides were added DAPI (Solarbio, Beijing, China) staining nuclei for 5 minutes at room temperature. Images were collected using the Zeiss Confocal Laser Scanning Microscope (Carl Zeiss, Oberkochen, Germany).

PicoGreen staining

PicoGreen staining was performed using Quant-iT Pico-Green dsDNA reagent kits from Thermo Fisher Scientific. Cells were fixed in 4% paraformaldehyde for 30 minutes at room temperature and permeabilized with 0.4% Triton X-100 in PBS for 30 minutes. PicoGreen was diluted with a cell culture medium at a dilution ratio of 1: 500 at 37°C for one hour. Fluorescence images were collected using the Zeiss Confocal Laser Scanning Microscope 800 (Carl Zeiss, Oberkochen, Germany). DAPI was used to mark nuclei.

Enzyme-linked immunosorbent assay (ELISA)

Detection of cytokines CCL5 and CXCL10 in cell supernatants was measured by ELISA Kit (Proteintech, Chicago, USA, Catalog # KE00093, KE00128). Culture media was collected after 72h treatment of PARPi. Assays were performed following manufacturers' protocols. And absorbance was measured at 450 nm with the

correction wavelength set at 630 nm using an Infinite®200PRO microplate spectrophotometer (Tecan, Salzburg, Austria).

Supplemental Tables

Supplemental table 1. Information of cGAS-STING pathway genes.

Gene symbol	Gene id	Source
CGAS	115004	PMID: 27648547
STING1	340061	PMID: 27648547
IRF3	3661	PMID: 27648547
TBK1	29110	PMID: 27648547
NFKBIA (IKBA)	4792	PMID: 27648547
ASCC1 (p50)	51008	PMID: 27648547
RELA (p65)	5970	PMID: 27648547
CHUK (IKK1)	1147	PMID: 27648547
NFKB1	4790	PMID: 27648547
IFN1	3438	PMID: 27648547
IL6	3569	PMID: 31358977
TNF	7124	PMID: 31358977
IKBKB (IKKB)	3551	PMID: 31358977
IFNB1	3456	PMID: 31358977
CCL5	6352	PMID: 30589644
CXCL10	3627	PMID: 30589644
CXCL8 (IL8)	3576	PMID: 31799772
IFNA1	3439	KEGG
IFNA2	3440	KEGG
IFNA4	3441	KEGG
IFNA5	3442	KEGG
IFNA6	3443	KEGG
IFNA7	3444	KEGG
IFNA8	3445	KEGG
IFNA10	3446	KEGG
IFNA13	3447	KEGG
IFNA14	3448	KEGG
IFNA16	3449	KEGG
IFNA17	3451	KEGG
IFNA21	3452	KEGG

Supplemental table 2. Marker genes from the dataset of GSE131907.

Class	Cell types	Genes	PMID
9 cell lineages	Epithelial cells	EPCAM, KRT19, CDH1, KRT18	28474673, 31067475
	T lymphocytes	CD3D, CD3E, CD3G, TRAC	28475900, 31209336
	B lymphocytes	CD79A, IGHM, IGHG3, IGHA2	31712411, 30523328
	Myeloid cells	CD68, MARCO, FCGR3A (CD16), LYZ	28475900, 29967419
	NK cells	NCAM1 (CD56), NKG7, GNLY, KLRD1	28475900, 31477722
	MAST cells	KIT, MS4A2,GATA2	30979687
	Fibroblasts	DCN, COL1A1, COL1A2, THY1	31209336, 29198524
	Endothelial cells	PECAM1, CLDN5, FLT1, RAMP2	30674341, 21460247, 23355623
	Oligodendrocytes	OLIG1, OLIG2, MOG, CLDN11	29615592, 26628089
A subset of Epithelial cells	AT1	AGER	30554520
	AT2	SFTPC, LAMP3	30554520
	Club	SCGB1A1	30554520
	Ciliated	FOXJ1, RFX2	30554520
A subset of Endothelial cells	Tip-like ECs	RAMP3, RGCC, ADM	29449267
	Stalk-like ECs	SELP, ACKR1	29449267
	Lymphatic ECs	CCL21, LYVE1	29449267
	EPCs	TYROBP, C1QB	29449267
	Tumor ECs	HSPG2, INSR, VWA1	29988129, 30559346, 10629090
A subset of Fibroblasts	COL13A1+ matrix FBs	COL13A1, TCF21, ITGAB, CXCL14, NPNT	29590628
	COL14A1+ matrix FBs	COL14A1, GSN, PI16, CYGB, PRRX1	29590628
	Myofibroblasts	ACTA2, MYH11, TAGLN, ACTG2, MYLK	29590628
	Smooth muscle cells	CNN1, SYNPO2, CRYAB, DES	28564607
	Mesothelial cells	UPK3B, MSLN, CALB2, WT1	29590628
	Pericytes	RGS5, CSPG4, ABCC9, KCNJ8	28564607
	Perivascular FB-like cells	CYP1B1, APOD	29443965
	Lipofibroblast	FABP4, FABP5, PPARG	29590628
A subset of Myeloid cells	Monocyte	CTSS, FCN1, S100A8, S100A9, LYZ, VCAN	29967419
	Macrophage	LGMN, CTSB, CD14, FCGR3A	29967419
	mo-lineage	MAFB, MAF, CX3CR1, ITGAM, CSF1R	28257233
	Alveolar-Mac	MARCO, FABP4, MCEMP1	28257233
	Anti-inflammatory	CD163, APOE, SEPP1, C1QA, C1QB, C1QC	27381735, 21350196, 26053663, 22523386
	Pro-inflammatory	CXCL8, IL1B	25339958
	Cycling	STMN1, MKI67, TOP2A, CDK1	29967419

	DC	CLEC10A, CD1C, CLEC4C, PTCRA, CCR7, LAMP3	28475900
A subset of DCs	CD1c+ DCs (LCs)	CD1C, ITGAX	24744755
	CD141+ DCs	CLEC9A, XCR1	24744755
	CD207+CD1a+ LCs	CD207, CD1A	24744755
	Activated DCs	CCR7, LAMP3	17312119
	pDCs	IL3RA, CLEC4C	28428369
	CD163+CD14+ DCs	CD14, CD163	31474513
A subset of B lymphocytes	GC B cells in the DZ	STMN1, AICDA, MKI67, BIRC5	30104629
	GC B cells in the LZ	LMO2, BCL2A1	30104629
	GrB-secreting cells	GZMB	21808264
	Follicular B cells	MS4A1, HLA-DRA	29988129
	MALT B cells	JCHAIN, IGHA1	29988129
	Plasma cells	IGHG1	29988129
A subset of T/NK cells	CD4+ T	Il7R, CD4	14662907, 28475900
	CD8+ T	CD8A, CD8B	28475900
	Naïve	TCF7, SELL, LEF1, CCR7	29942094
	Exhausted	LAG3, TIGIT, PDCD1, HAVCR2	29942094
	Cytotoxic	IL2, GZMA, GNLY, PRF1, GZMB, GZMK, IFNG, NKG7	29942094
	Treg	IL2RA, FOXP3, IKZF2, TGFB1, TGFB3, TGFBI, TGFBR1	29942094, 28474673
	T follicular helper	MAF, CXCR5, PDCD1, CXCL13	28265271, 28570278
	T helper 17	IRF4, CREM, NR4A2	21381156, 27680869, 23437182
	T helper 1	STAT4, IFNG, IL12RB2	24987392, 21685955
	T helper 2	GATA3, STAT6, IL4	24987392
	gamma delta T	TRDC, TRGC2, TRGC1	31118283
	NK	XCL1, FCGR3A, KLRD1, KLRF1	31477722

Supplemental Figures

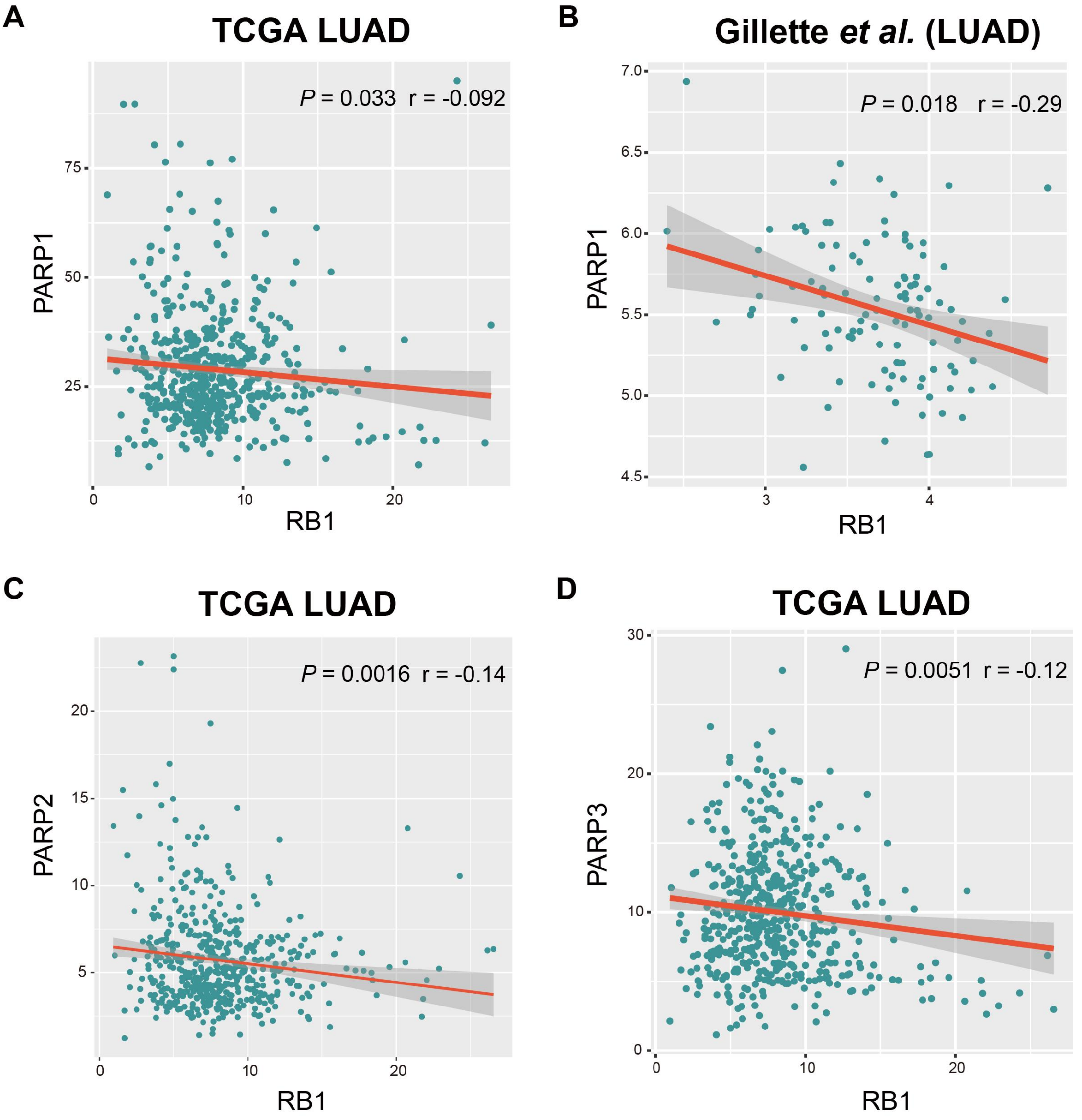

Supplemental figure 1. Correlation of the expression of *RB1* and *PARP1*.

(**A-D**) Pearson correlation analysis of the expression of *RB1* and *PARP1* in TCGA and Gillette *et al.* datasets.

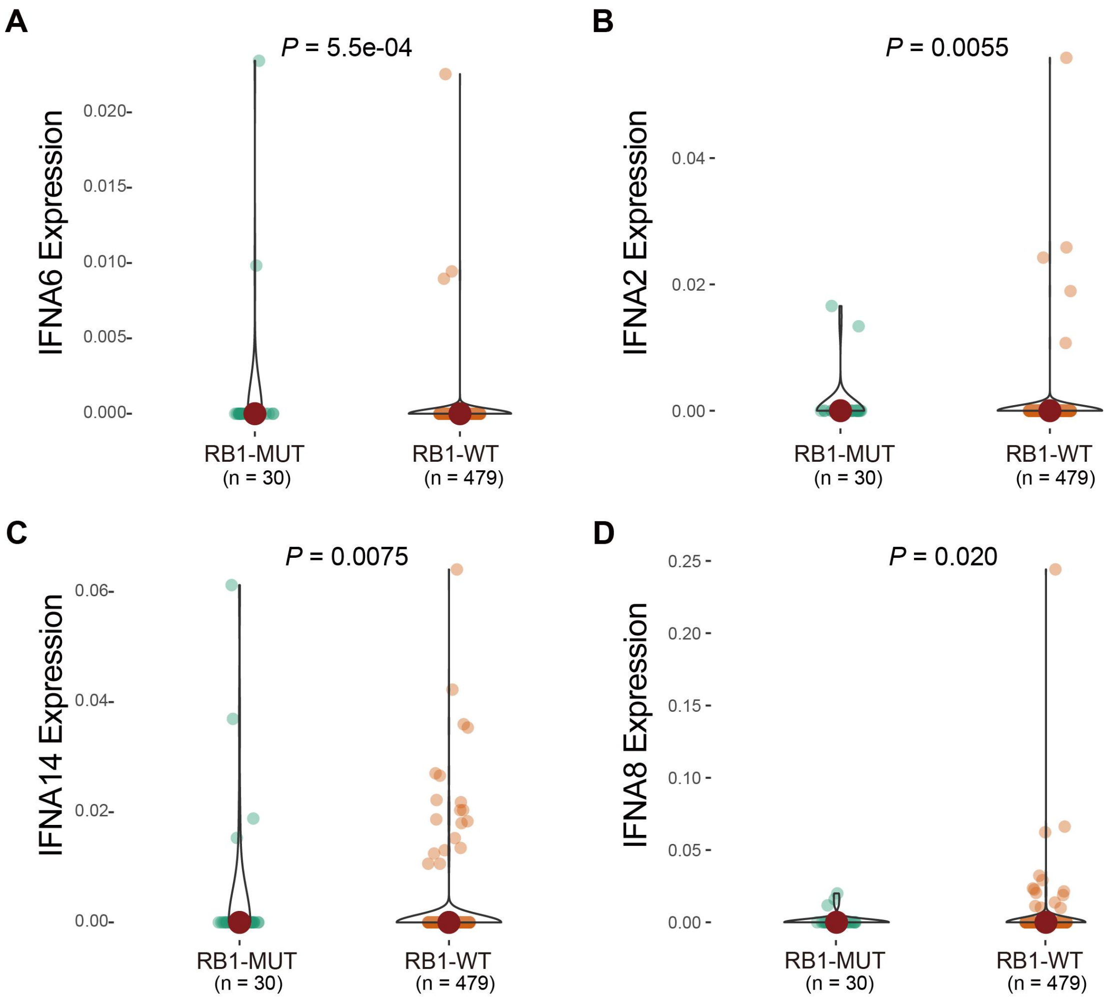

Supplemental figure 2. Differential expression of four type I interferon genes.

(**A**-**D**) Differential expression of type I interferon genes (*IFNA6*, *IFNA2*, *IFNA14* and *IFNA8*) between *RB1* mutant and *RB1* wild-type samples in TCGA LUAD using one-sided Wilcoxon rank-sum test.

A

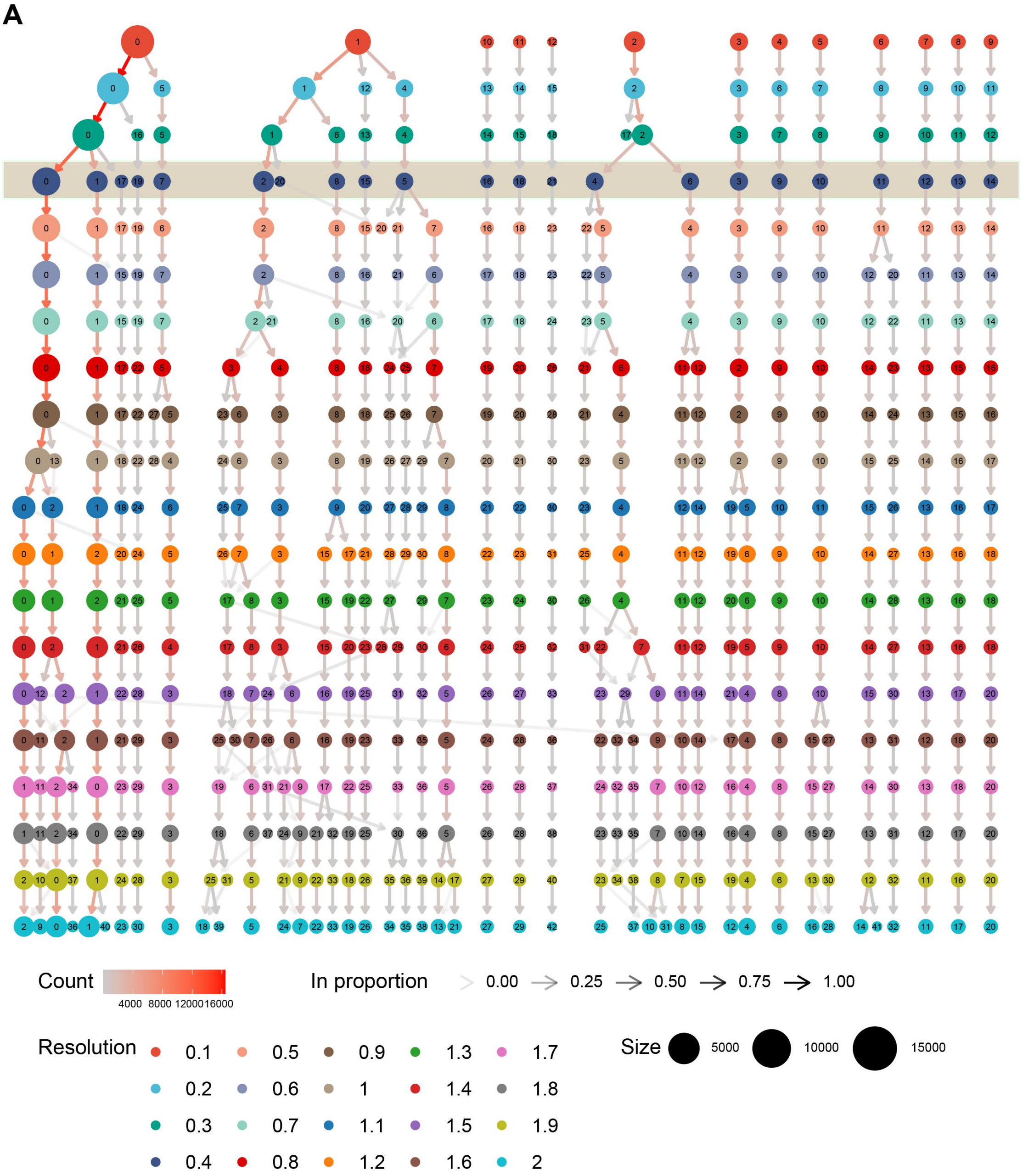

Supplemental figure 3. The clustering tree at multiple resolutions.

(**A**) The size of node denotes the number of cells in a cluster. The color of node represents the resolution value. Edges are colored according to the number of cells they represent and the transparency shows the incoming node proportion.

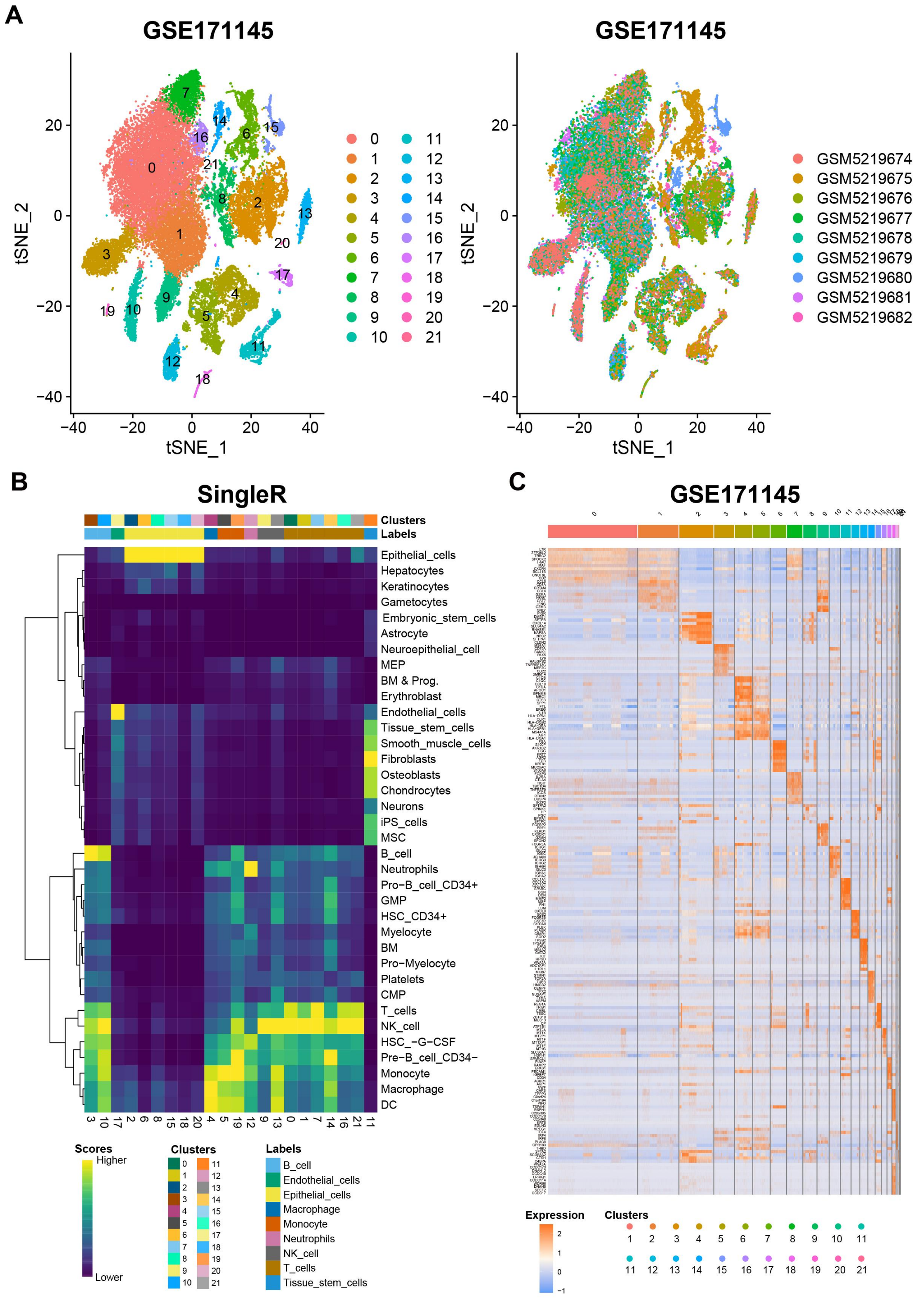

Supplemental figure 4. Identification of cell clusters and cell type annotation in GSE171145.

(A) The tSNE visualization of LUAD cells colored by cell clusters after corrected

batch effect by “Harmony” (left) and patients (right) in GSE171145. **(B)** The initial assignment scores for each cluster (columns) to each reference label (rows) by “SingleR”. **(C)** The top 10 differentially expressed genes for each cluster.

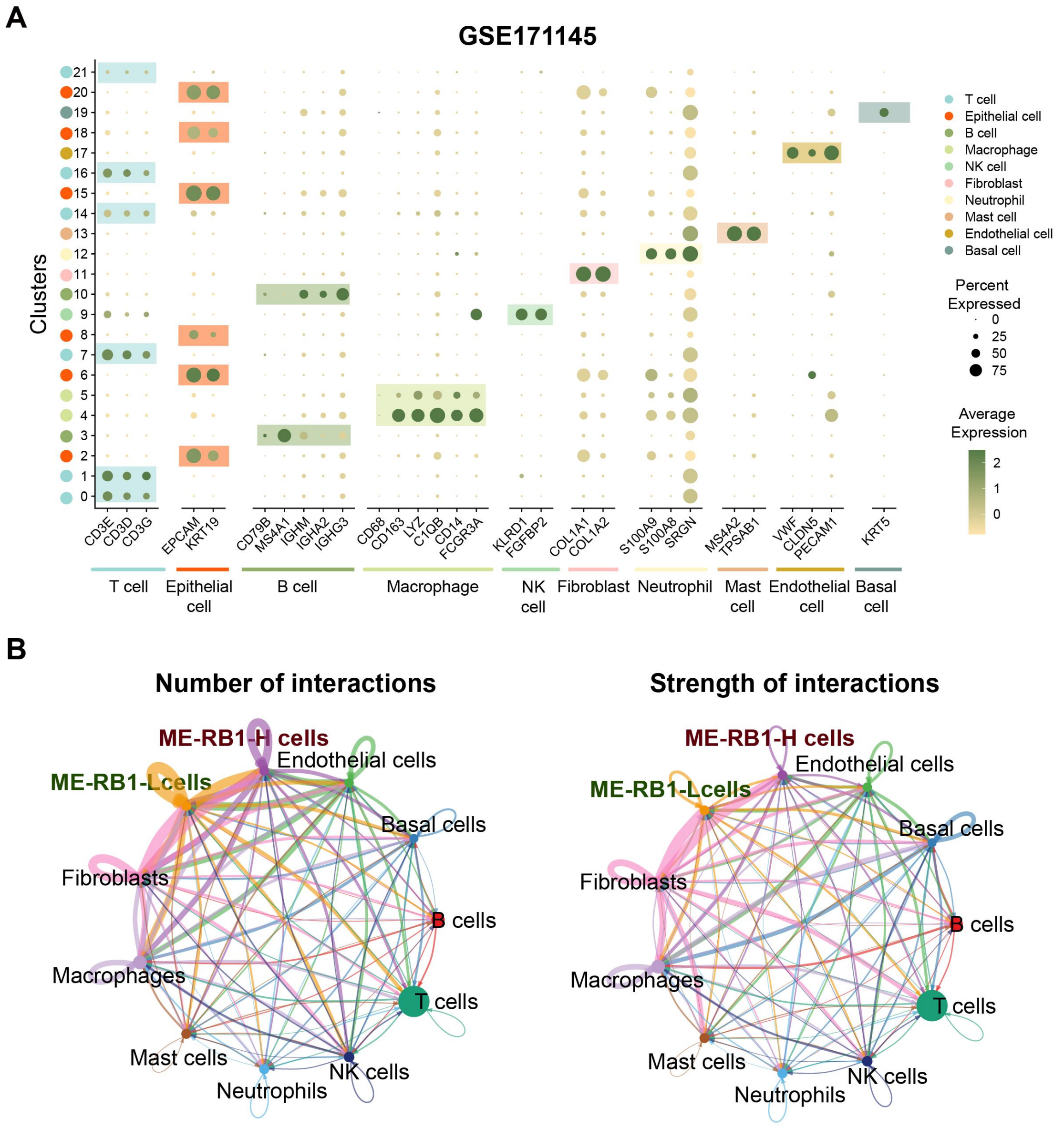

Supplemental figure 5. Expression of canonical marker genes and communications among cell types in GSE171145.

(A) The expression of canonical cell type marker genes in each cell cluster. **(B)** Numbers and strength of interactions among ME-RB1-L, ME-RB1-H, and other cell types in GSE171145.

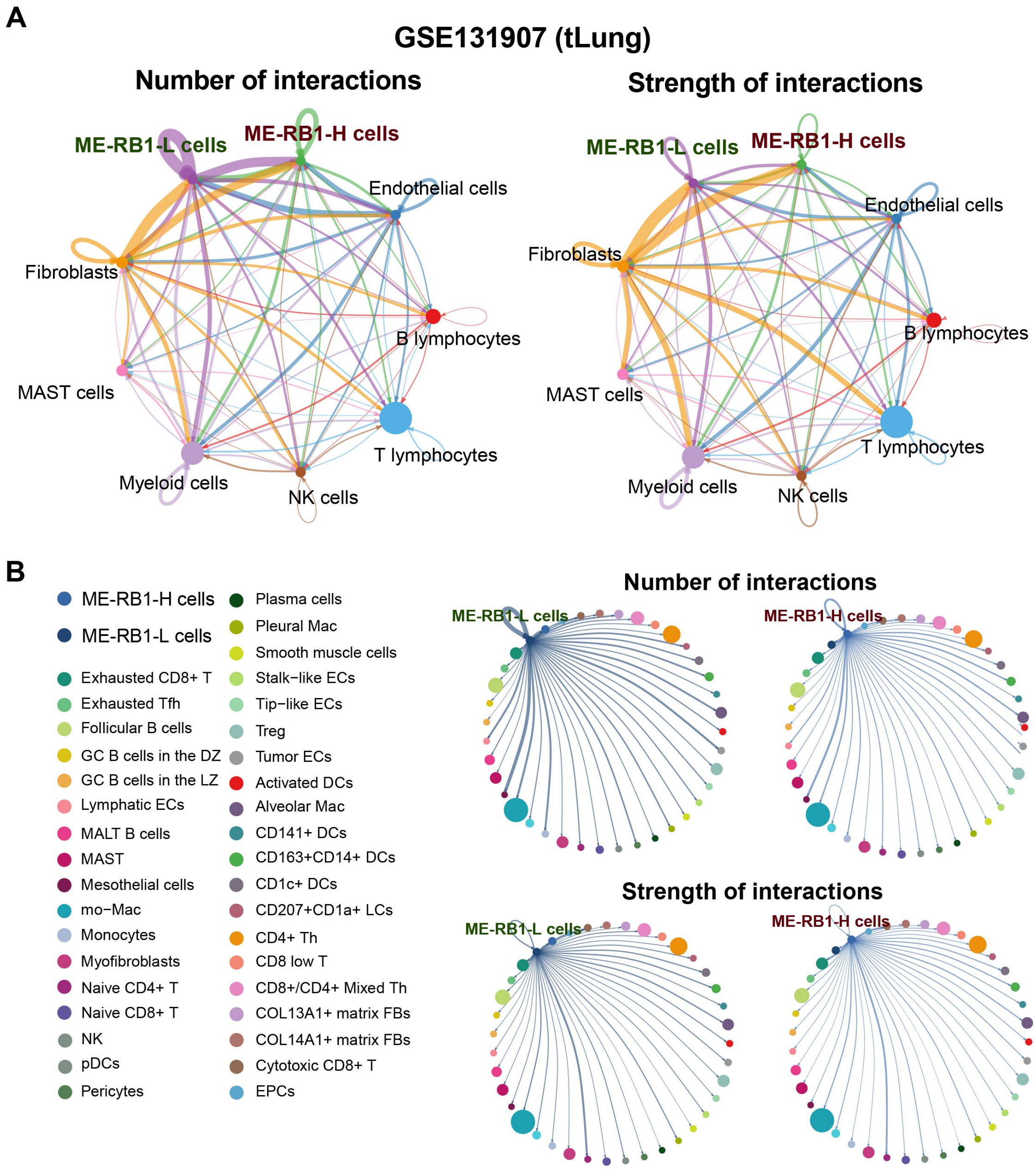

Supplemental figure 6. Analysis of communications among cell types in GSE131907.

(A) Numbers and strength of interactions among ME-RB1-L, ME-RB1-H, and other cell types in GSE131907 (tLung). **(B)** Numbers and strength of interactions among ME-RB1-L, ME-RB1-H, and other cell subtypes in GSE131907 (tL/B).

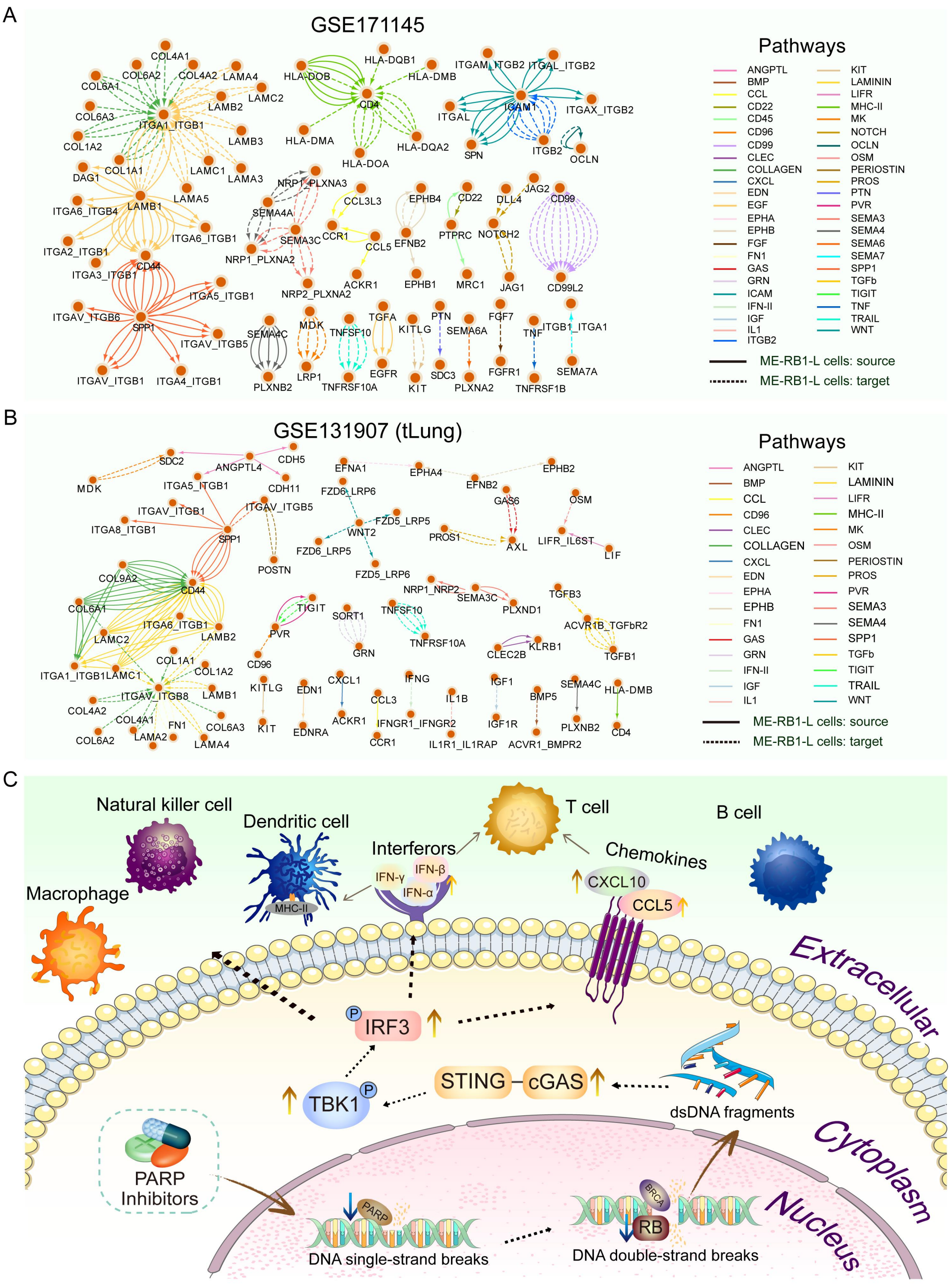

Supplemental figure 7. Schematic diagram of therapeutic mechanism.

(**A** and **B**) Specific communications in ME-RB1-L cells compared with ME-RB1-H cells in GSE171145 and GSE131907 (tLung) datasets. Edges represent the interactions between ME-RB1-L cells and other cell types, the color of edges means

the signaling pathways which the interactions belong to, and the type of edges represents the ME-RB1-L cells as senders or receivers. (**C**) The model of the activation of immune response via PARPis.

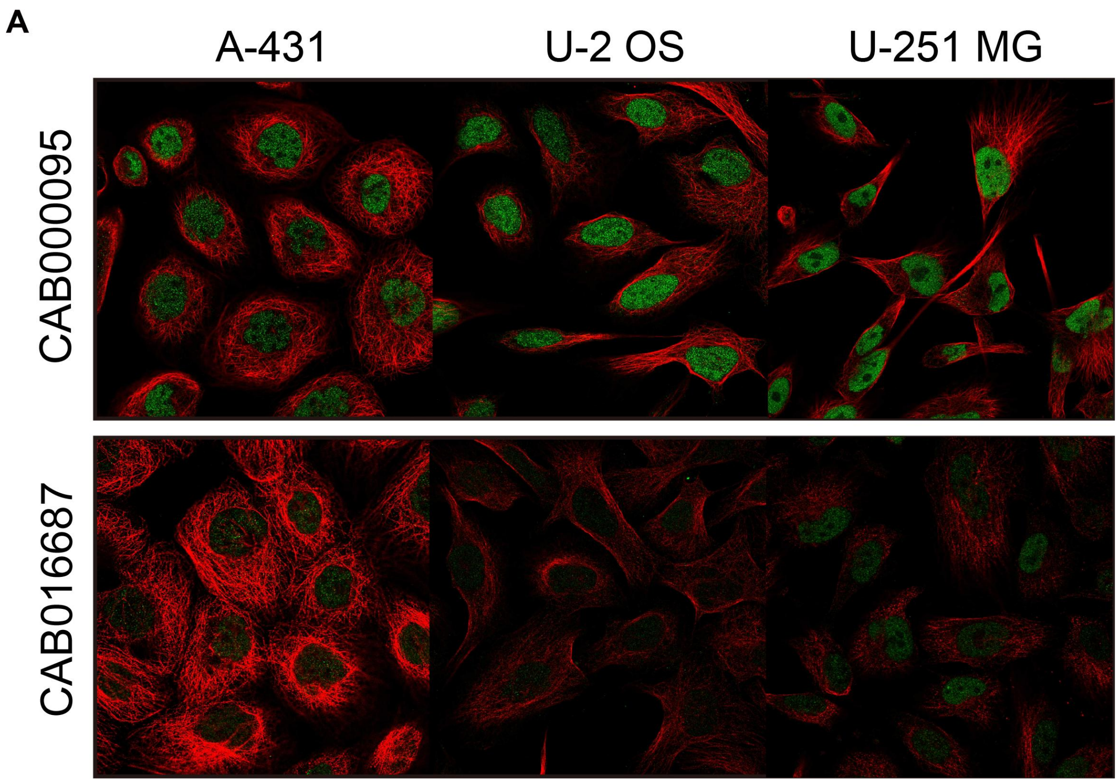

Supplemental figure 8. ***RB1*** **localizes in the nucleoplasm.**

(A) *RB1* localizes in the nucleoplasm, indicated by staining on A-431, U-2 OS, and U-251 MG cells targeted by CAB000095 and CAB016687 antibodies. Location information of *RB1* was obtained from The Human Protein Atlas database (https://www.proteinatlas.org/).
